# Legacy effects of historical gold mining on floodplains of an Australian river

**DOI:** 10.1007/s10653-024-02003-5

**Published:** 2024-06-13

**Authors:** Francesco Colombi, Aleicia Holland, Darren Baldwin, Susan Lawrence, Peter Davies, Ian Rutherfurd, James Grove, Jodi Turnbull, Mark Macklin, Greg Hil, Ewen Silvester

**Affiliations:** 1 Department of Environment and Genetics, School of Agriculture, Biomedicine and Environment, La Trobe University, Albury/Wodonga Campus, Wodonga, VIC 3690 Australia; 2 School of Agricultural, Environmental and Veterinary Sciences, Charles Sturt University, Thurgoona, NSW 2640 Australia; 3 River and Wetlands, Thurgoona, NSW Australia; 4 Department of Archaeology and History, La Trobe University, Bundoora, VIC 3086 Australia; 5 School of Geography, Faculty of Earth and Atmospheric Science, University of Melbourne, 22 Bouverie Street, Melbourne, VIC 3001 Australia; 6 School of Geography and Lincoln Centre for Water and Planetary Health, College of Science, University of Lincoln, Lincoln, Lincolnshire LN6 TS UK

**Keywords:** Gold mining, Floodplain sediments, Arsenic, Rivers, Victoria

## Abstract

The gold rush at the end of the nineteenth century in south-eastern Australia resulted in the mobilization and re-deposition of vast quantities of tailings that modified the geomorphology of the associated river valleys. Previous studies of contamination risk in these systems have either been performed directly on mine wastes (e.g., battery sand) or at locations close to historical mine sites but have largely ignored the extensive area of riverine alluvial deposits extending downstream from gold mining locations. Here we studied the distribution of contaminant metal(loids) in the Loddon River catchment, one of the most intensively mined areas of the historical gold-rush period in Australia (1851–1914). Floodplain alluvium along the Loddon River was sampled to capture differences in metal and metalloid concentrations between the anthropogenic floodplain deposits and the underlying original floodplain. Elevated levels of arsenic up to 300 mg-As/kg were identified within the anthropogenic alluvial sediment, well above sediment guidelines (ISQG-high trigger value of 70 ppm) and substantially higher than in the pre-mining alluvium. Maximum arsenic concentrations were found at depth within the anthropogenic alluvium (plume-like), close to the contact with the original floodplain. The results obtained here indicate that arsenic may pose a significantly higher risk within this river catchment than previously assessed through analysis of surface floodplain soils. The risks of this submerged arsenic plume will require further investigation of its chemical form (speciation) to determine its mobility and potential bioavailability. Our work shows the long-lasting impact of historical gold mining on riverine landscapes.

**Supplementary Information:**

The online version contains supplementary material available at 10.1007/s10653-024-02003-5.

## Introduction

Historical mining activities around the world have produced enormous quantities of waste materials in river catchments (Macklin et al., 2023). In South-Eastern Australia, gold mining was largely limited to the period 1851–1914. During this period, Central Victoria was one of the most important mining regions in terms of gold production (total production of ~ 2500 t Au) (Phillips & Hughes, 1996). Consequently, historical gold mining activity in Victoria has left a significant legacy of mine wastes (Lawrence & Davies, 2019; Davies et al., 2018a, 2018b; Lawrence & Davies, 2014; Lawrence et al., 2016). Mine wastes can include: solid, liquid and/or gaseous wastes of the mining and metallurgical processes that can have adverse effects on the surrounding environment, particularly rivers and their associated floodplains (Clement et al., 2017; Frohne et al., 2014; Lewin & Macklin, 1987; Lottermoser, 2010; Macklin et al., 2006; Onnis et al., 2023; Raab & Raab, 2010) Gold mine wastes may also contain elevated levels of metal(loids) such as arsenic (As) and mercury (Hg) (Alpers, 2017; Wongsasuluk et al., 2021). Arsenic and Hg contamination in Victorian catchments has previously been reported in surface waters, creek bed sediments and freshwater lake sediments (Churchill et al., 2004; Lintern et al., 2020; Sultan, 2007; Sultan & Dowling, 2006).

Arsenic is often associated with the accessory minerals in gold-bearing ores (Craw & Bowell, 2014; O'Day, 2006). Once exposed to atmospheric conditions during metallurgical operations, As may become mobile at mine sites as a result of multiple (and often related) processes, including: size reduction (i.e., crushing and grinding), oxidation and dissolution of As-bearing sulfide minerals (e.g., arsenopyrite, FeAsS) (Craw & Bowell, 2014; Liu et al., 2022). These processes can result in the release of arsenic, as a component of mine tailings, into creeks and streams (Rae, 2001; EPA, 2016, Faria et al., 2023). As-containing particles can be further transported through rivers and streams as either bedload, suspended sediments or as dissolved species in the water column, where they can be stored in river bed sediments or as floodplain alluvial deposits from overbank events (Byrne et al., 2012). Further biogeochemical cycling of As stored in these deposits will control its mobility and potential toxicity (Ciszewski & Grygar, 2016; Du Laing et al., 2009; Macklin et al., 2006; Tokalioğlu et al., 2003). Abraham and co-workers found increased levels of As in mine sediments and suggested that As can also be used as a marker of presence of mine sediments (Abraham et al., 2018).

Mine waste sediments deposited on floodplains are also often identified by different macroscopic (colour, stratigraphic units) and microscopic (texture) physical characteristics compared to the underlying and relatively older strata (Allan James et al., 2020; Clement et al., 2017). An important variable in assessing metal(loid) mobility from mine wastes is the grain size distribution. The metal(loid) content in mine wastes, soils and sediments has been shown to vary with grain size, typically with an inverse trend (Doherty et al., 2022; Lai et al., 2013). Consequently, due to higher available surface area, higher metal(loid) concentrations are generally associated with the finest size fraction (i.e., clay-silt fraction; < 63 µm) (Dennis et al., 2003).

The contamination of floodplains with metal(loid)-rich waste often shows a general decrease in metal(loid) concentrations downstream from the source area (Johnston et al., 2020; Miller, 1997). The perturbation of the riverine landscape associated with mining continues well after the mining operation is abandoned, often due to failure of tailing dams (Hatje et al., 2017; Macklin et al., 2003, 2023) or by episodic flood events which can remobilise contaminated sediment deposits (Ciszewski & Grygar, 2016; Clement et al., 2017; Crawford et al., 2022; Foulds et al., 2014). Consequently, catchments draining mine-affected areas can have metal(loid) concentrations orders of magnitudes higher than average background levels (Foulds et al., 2014; Macklin et al., 2006).

In Victoria, mine sites and their surrounds have been found to be enriched in metal(loid) concentrations compared to areas not affected by past mining activities (Abraham et al., 2018; Churchill et al., 2004; Davies et al., 2015; Martin et al., 2016; Noble et al., 2010; Sultan, 2007). Similarly, the health effects associated with mining in Victoria have been previously assessed (Abraham et al., 2018; Pearce et al., 2010), however these studies have been restricted to areas immediately around mine sites. Despite metal(loid) contamination in Victoria being previously investigated, limited information is available on the effects past mining activities have on the chemical and physical characteristics of downstream floodplain sediments in affected catchments. This study aims to evaluate the effects of historical gold mining on Victorian river catchments with respect to changes to sediment characteristics and the distribution of metal(loids) in floodplain sediments. In addition, a hotspot analysis was conducted to compare surface contamination with potential subsurface accumulation of metal(loid)s within Victorian catchments. This study focused on the Loddon River catchment, which included some of the most important and productive goldfields of the gold rush period. More than 80 million cubic metres of mining sediments were mobilized within this catchment during the gold rush and it is one of the most impacted river basins in south-eastern Australia (Davies et al., 2018a, 2018b).

## Materials and method

### Study area

**Fig. 1:**
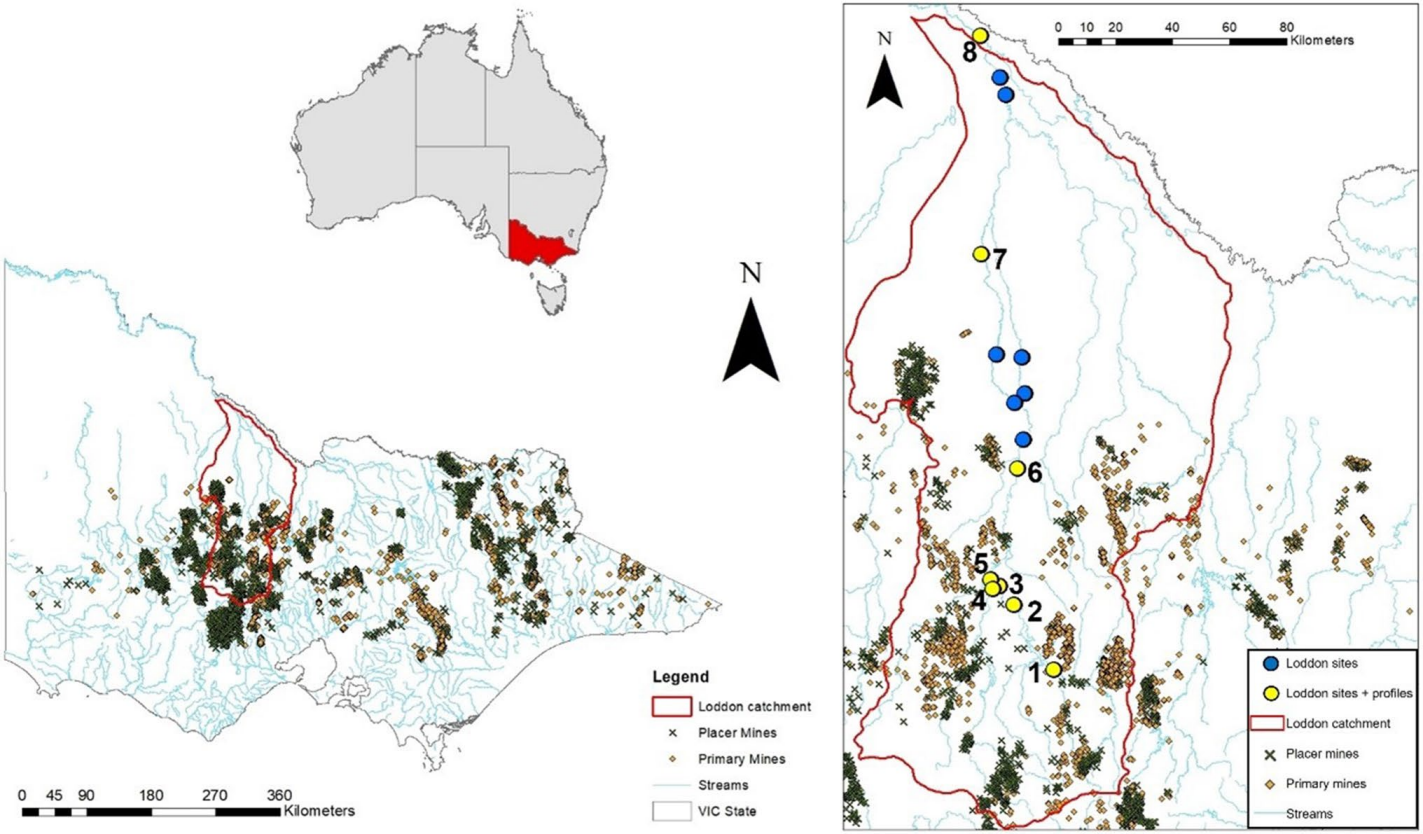
Location of study sites. **a** Location of the Loddon River catchment within Victoria. Also shown are VICMine data for locations of primary and placer (secondary, alluvial) gold mines for the period 1864–1960 (from: State Victoria, Earth Resources Development Division 2002)). **b** Study sites for this work: 1 = Newstead; 2 = Baringhup; 3 = Back Eddington; 4 = Tullaroop Creek; 5 = Eddington; 6 = Bridgewater; 7 = Boort-Yando; 8 = Benjeroop) and mining locations within the Loddon River catchment

The Loddon River is the second longest river in Victoria and a tributary of the Murray-Darling Basin, extending approximately 400 km from its headwaters near Castlemaine to the Murray River (Fig. 1) (Leevers 2015). The Loddon catchment covers a total area of approximately 15,000 km^2^, corresponding to half of the North Central region of Victoria (Fig. 1). The average annual rainfall ranges from 800 mm yr^−1^ in the relatively high-relief southern part of the catchment to around 300 mm yr^−1^ in the north, indicating a semi-arid climate, with an average annual discharge of about 201 GL/yr (Leevers 2015). Anabranching systems linked by smaller tributary channels form floodplains within the catchment where the main river’s gradient significantly decreases, around 200 km downstream. In contrast, the headwaters where mining activity have taken place tend to have far less extensive floodplains (Leevers 2015). The physiography and geomorphology of this hydrographic basin has been deeply altered by human activities during the last two centuries, through gold mining and the subsequent expansion of land clearing for agriculture after the decline of the gold rush (Abernethy et al., 2004; Flatley & Rutherfurd, 2019).

### Field survey

Samples of sediment profiles were collected in April, June and October 2018, from eight sites along the Loddon River and one of its tributaries (Tullaroop Creek site). The sampling locations included sites along the river downstream of potential source areas of mine pollution, including both primary and placer deposits that are widely distributed in Victoria, particularly across the Loddon catchment (from VICMINE dataset, State Victoria, Fig. 1). The investigated sites were chosen through the identification of possible areas where deposition of anthropogenic sediments may have occurred, using LIDAR DTM (Digital Terrain Models) imagery in combination with historical archaeological background research of the area. Sampling locations were identified using Global Positioning System (GPS) and maps were produced with ArcMap Software (version 10.6.1).

A detailed in-field sediment sampling methodology (from the surface to between 1 and 3 m depth) was undertaken using a portable X-ray fluorescence (p-XRF) spectrometer (DELTA, Olympus). XRF measurements were taken every 10 cm on freshly exposed riverbank soils. A total of 107 samples were collected from the same locations as XRF measurements; these were stored in sealed plastic bags on ice, transported to the laboratory and frozen (− 20 °C). The p-XRF device was calibrated daily against the certified standard materials (NIST2711a and NIST2710a). The elements measured by p-XRF were As, Fe, Mn, Cr, Cu Zn and Pb.

### Soil analysis

For the determination of soil moisture content, samples (between 4 and 7 g) were dried at 105 °C to constant weight in a hot air oven, transferred to a dry desiccator to cool and then weighed. To determine the loss on ignition (LOI) content, a proxy for total organic carbon (TOC), oven-dried samples were transferred to a muffle furnace and heated at 500–550 °C for 2 h to combust organic matter and then transferred to 105 °C hot air oven for 30 min. After cooling, the samples were re-weighed and the percentage LOI calculated.

Dry sieving using 250 μm and 63 μm sieves was performed to obtain the three size fractions of interest: medium sand (> 250 μm), fine to very fine sand (250–63 μm) and silt/clay (< 63 μm) (reported as mean ± 1SD). The different proportions of each sediment fraction was classified using ternary diagrams according to the Shepard’s Classification System (Shepard, 1954). Samples were also analysed for particle size distribution (texture) in the range 10–90 μm using a LS 13 320 Laser Particle Sizing (LPS) Analyzer.

For metals and metalloid analysis (reported as mean ± 1SD) sediment was acid digested using a standard microwave digestion method (Method 3050B; US Environmental Protection Agency (Agency & Agency-Usepa, 1996)). Air dried subsamples of 0.1 g were weighed into 10 mL Teflon microwave digestion vessels, treated with 2.5 mL of concentrated HNO_3_ (OPTIMA grade; 69%) and left overnight. The next day 0.5 mL H_2_O_2_ (30%) was added and the samples digested in a MARS 6 Microwave with the following temperature profile: ramp to 200 °C (20 min); controlled at 200 °C for 15 min; cooled to < 70 °C (15 min). After cooling, digested samples were made up to 50 mL with Milli-Q water. Each digestion batch included two reagent blanks as contamination controls. One mL of the digested supernatant was diluted with 4 mL of Milli-Q water in a 10 mL tube with the addition of 0.25 mL of 0.1% Ni(NO_3_)_2_ as an ionisation suppressant.

Total arsenic concentrations were measured by graphite furnace atomic absorption spectroscopy (GF-AAS) using a GTA-120 graphite tube atomizer (Varian) coupled with a AAS 240 FS spectrometer (Varian) and with a single element As-cathode Ultra-AA lamp. In each analysis run a 3-point calibration curve over the range 0–75 ppb As was generated automatically by ratio mixing of a 50 ppb As standard (prepared from: 1000 ppm As certified standard; Sigma Aldrich) and a 2% HNO_3_ blank. Each analysis batch included check standards with concentrations between 2 and 50 ppb As for drift detection and sample blanks for zero correction.

Acid digests for two riverbank soil profiles (Back Eddington and Bridgewater) were also analysed by inductively coupled plasma-mass spectrometry (ICP-MS) at the Environmental Futures Research Institute (Griffith University) for a suite of 19 elements, including major and trace metals, for the determination of total metal concentrations in different grain-size fractions. River sediment certified reference materials (SLRS-6 CRM 1; SLRS-6 CRM 2) yielded recoveries of 99.6–104.5% and 82.5–109.9% for major and trace elements, respectively.

### Statistical analysis

The dataset was categorised into two groups, anthropogenic sediments and original floodplain sediments. The distinction between groups was based on the macroscopic (e.g., colour), textural and stratigraphic characteristics of the riverbanks sampled, i.e., in-field assessment of the contact between anthropogenic sediments and the original floodplain deposits (Supplementary Information, Figures S1–S2). The two-sample Kolmogorov–Smirnov test (K–S test), assuming a significant level α = 0.05, was used to test the null hypothesis that the two groups belong to the same distribution. Differences in sediment composition between the two groups (anthropogenic and original floodplain sediments) were investigated with non-metric statistical techniques: analysis of similarities (ANOSIM), similarity percentages (SIMPER) and multivariate principal component analysis (PCA). These analyses were performed using package PRIMER-7 (Baldwin et al., 1998; Clarke, 1993) and the ggplot2 package in R (Wickham, 2016). The variables analysed included: Fe, Mn, Cr, Cu, As, Zn from p-XRF analysis, the three size fractions (> 250 μm, 250–63 μm, < 63 μm; expressed as percent) and LOI (%). A second evaluation included Mg, Al, Cr, Mn, Fe, Cu, As, Se, Mo, Cd, Sb, W, Pb, U analysed by ICP-MS along with LOI (%) and the three size fractions (%), as variables. ANOSIM and PCA analyses were derived from a resemblance matrix using the Euclidean distance. SIMPER was performed using Bray–Curtis similarities of the two groups.

Arsenic concentrations from surface samples obtained from mineral licenses expired prior to 1965 (GeoVic database) were used to evaluate arsenic clustering around Victoria. Natural log-transformed data was used for spatial analysis of arsenic soil contamination from this dataset, which includes thousands of sites across the state. This dataset was used as the input feature for the kernel density realized with ArcMap 10.6.1. Kernel density tools have been used to estimate the density of point features (arsenic point analysis) in the surrounding area. Optimized hot spot analysis works by looking at each sample within the context of neighbouring sampling points. To be statistically significant, a hot spot must have a high value and be surrounded by other samples with high values. Accordingly, a hot spot represents its local area rather than a single point. The transformation of a dot pattern into a continuous surface makes the kernel density map an easier way to visualise possible pollution hotspots.

## Results

### Sediment lithology and grain particle size

Riverbank sediment texture varied from sandy clay loam (e.g., Newstead) to loam (e.g., Boort-Yando) and was characterized by both vertical and lateral heterogeneity (Table 1). From upstream, where maximum mine waste sedimentation occurred (Newstead, 2 m; Supplementary Figure S1), we observed a gradual decrease in the thickness of the yellow-coloured layers identified here as anthropogenic sediments. Anthropogenic deposits were not identified at the two most downstream sites: Boort-Yando and Benjeroop (Supplementary Figure S1). Apart from these two sites, all other sites exhibited a sharp change in colour that identified the stratigraphic contact between two different sedimentary deposits (Figures S1–S2).

**Table 1:** Summary of analytical results (p-XRF) from all eight riverbank profiles, subdivided into: anthropogenic sediments and original floodplain deposits, Loddon River catchment

	Min	Max	Mean (± 1SD)
*LOI (%)*			
Anthropogenic sed	0.69	13.46	3.77 (± 2.71)
Original floodplain	0.31	6.38	3.91 (± 1.61)
*Silt/Clay (%)*			
Anthropogenic sed	0.13	75.91	37.74 (± 17.6)
Original floodplain	5.02	56.8	27.87 (± 13.90)
*Fine sand (%)*			
Anthropogenic sed	4.05	80.65	43.93 (± 14.85)
Original floodplain	29.12	64.16	45.53 (± 8.11)
*Sand (%)*			
Anthropogenic sed	b.d.l	94.14	10.96 (± 15.42)
Original floodplain	b.d.l	52.15	19.83 (± 16.81)
*Fe*			
Anthropogenic sed	8008	40,781	21,776 (± 5544)
Original floodplain	4462	34,962	21,927 (± 7749)
*Mn*			
Anthropogenic sed	b.d.l	668	227.75 (± 110.76)
Original floodplain	2	780	248.71 (± 181.77)
*Cr*			
Anthropogenic sed	b.d.l	110	56.39 (± 22.79)
Original floodplain	15	94	52.95 (± 15.21)
*As*			
Anthropogenic sed	4.1	312	47.60 (± 48.29)
Original floodplain	0.8	41.3	11.77 (11.19)
*Cu*			
Anthropogenic sed	b.d.l	16	3.87 (± 2.91)
Original floodplain	b.d.l	7	2.43 (± 2.27)
*Zn*			
Anthropogenic sed	19.9	65	41.28 (± 11.78)
Original floodplain	6	64.6	39.06 (± 16.00)
*Pb*			
Anthropogenic sed	10.9	36	20.15 (± 5.39)
Original floodplain	8.7	23.1	14.97 (± 3.67)

Anthropogenic sediments were characterized by a more variable particle size distribution and by a higher percentage of finer sediments compared to the underlying original floodplain sediments; this was particularly the case for the three uppermost sites (Newstead, Barringup, Back Eddington) but less evident in the remaining downstream sites (Figure S3). For example, at the Newstead site the silt/clay component of the anthropogenic sediments ranged over 0.1–68% compared to 6.8–19% for the original floodplain. Except for Back Eddington and the two most downstream sites (Boort Yando, Benjeroop), both the anthropogenic and the original floodplain sediments were enriched in fine/very fine sand (250–63 μm) with mean values of 44 ± 15% and 46 ± 8%, compared to the mean silt/clay size fraction, around 38 ± 18% and 28 ± 14%, respectively. The sand fraction of the anthropogenic sediments was composed of poorly sorted particles, mainly quartz characterized by low sphericity and angular shape. The presence of quartz sand characterized by a sharp morphology may be indicative of crushing in a stamp battery (Lottermoser, 2010). A similar texture was indeed observed in the sand fraction of a tailings heap sample collected in the upper part of the catchment, from the North British Mine at Maldon, Victoria (Supplementary Figure S4).

The particle size distribution (PSD) curves of the original floodplain deposits determined by laser particle size analysis showed a more uniform pattern compared to the PSD of the anthropogenic sediments (Supplementary Figure S5). By contrast, the Benjeroop site did not show any systematic difference in PSD curves through the soil profile, consistent with the absence of the contact. Loss on ignition (LOI) values for anthropogenic sediments and original floodplain deposits ranged from 0.7 to 13.5% and from 0.3 to 7.1%, respectively (Supplementary Figure S6); indicating that the organic matter content of both deposits from this area is quite variable. For the upper sites the LOI increases at the contact, consistent with a high organic pre-existent floodplain. For most downstream sites, including Tullaroop Creek, higher LOI values at the top of the profile suggest the re-establishment of an organic layer.

### Elemental composition

#### p-XRF

The average concentrations for metals and metalloids in riverbank sediment deposits, as determined via p-XRF, are summarized in Table 1 and expanded in Table S1. Arsenic in the anthropogenic sediments ranges from 4.1 to 312 mg/kg with a mean value of 48 ± 48. Significantly less As was measured in the original floodplain sediments (*p* < 0.001), with values ranging from 0.8 to 41 mg/kg (12 ± 11 mg/kg). Arsenic concentrations were comparable between p-XRF and GF-AAS (Supplementary Figure S7; R^2^ = 0.7), although GF-AAS concentrations were generally higher than p-XRF (up to 3% for some points measured).

The original floodplain sediments contained significantly less Pb than the anthropogenic sediments (p < 0.001), with values of 15 ± 4 mg/kg and 20 ± 5 mg/kg, respectively (Table 1-S1). The average concentrations of zinc (Zn) and copper (Cu) remained well below the ISQG low trigger values of 200 mg/kg and 65 mg/kg respectively, although Cu concentrations were significantly higher in the anthropogenic sediments compared to the original floodplain deposits (*p* = 0.009). Anthropogenic sediments contained chromium (Cr) varying from 0 to 110 mg/kg (56 ± 23 mg/kg), whereas the original floodplain sediments were characterized by Cr contents from 15 to 94 mg/kg (53 ± 15 mg/kg), a difference which was not significant (*p* > 0.01). All the investigated soil profiles except for Benjeroop contained single samples with Cr values higher than the ISQG low trigger value (80 mg/kg).

Floodplain sediments were enriched in iron (Fe) and manganese (Mn) compared to the anthropogenic sediments, although the differences were not significant (*p* > 0.01) (Table 1-S1). The iron concentration in the anthropogenic sediments and floodplain deposits displayed a similar variability, with values ranging from 8008 to 40,781 mg/kg (22,000 ± 6000 mg/kg) and from 4462 to 34,962 mg/kg (22,000 ± 8000 mg/kg), respectively. Floodplain sediments contained Mn concentrations as high as 780 mg/kg, with a mean value of 249 ± 182 mg/kg, whereas the concentrations of this metal in the anthropogenic sediments ranged from 0 to 668 mg/kg (228 ± 111 mg/kg).

#### ICP-MS

Back Eddington and Bridgewater river-bank deposits were further characterized for metal(loid)s content using ICP-MS (Table S2). Descriptive statistics for the metal contents of the two deposits analysed via ICP-MS are summarized in Table 2. Arsenic concentrations determined by ICP-MS were similar to that determined by GF-AAS (R^2^ = 0.9) and p-XRF (R^2^ of 0.7 and 0.9 for Back Eddington and Bridgewater, respectively). As observed for GF-AAS results, anthropogenic sediments were characterized by significantly more As than floodplain sediments (*p* < 0.001), with values of 42 ± 29 mg/kg and 4 ± 3 mg/kg, respectively. Unlike the results from p-XRF analyses, for these two particular sediments, metals such as Cu, Pb and Mn were all elevated in floodplain sediments (with average values of 106 (± 282), 17 (± 24) and 232 (± 280), respectively), compared to anthropogenic sediments (with average values of 16 (± 8) for Pb, 27 (± 63) for Cu and 190 (± 93) for Mn). Iron concentrations were higher in anthropogenic sediments, from 3010 to 33,120 mg/kg (19,000 ± 8100 mg/kg) with respect to floodplain sediments, from 1396 to 30,351 mg/kg (12,200 ± 9000 mg/kg). By contrast, aluminium (Al) concentrations were higher in floodplain sediments, from 911 to 42,976 mg/kg (14,300 ± 13,000 mg/kg) compared to anthropogenic sediments, which ranged from 2972 to 39,015 mg/kg (17,600 ± 280 mg/kg). Also, magnesium (Mg) was found in higher concentrations in floodplain sediments, with values from 85 to 5208 mg/kg (1700 ± 1500 mg/kg) than anthropogenic sediments, from 297 to 3844 mg/kg (190 ± 93 mg/kg).

**Table 2:** Summary of analytical results (ICP-MS) from two riverbank profiles (Back Eddington and Bridgewater), subdivided into: anthropogenic sediments and original floodplain deposits, Loddon River catchment

	Min	Max	Mean (± 1SD)
*LOI (%)*			
Anthropogenic sed	2.34	9.38	3.27 (± 1.74)
Original floodplain	0.31	6.12	2.50 (± 2.15)
*Silt/Clay (%)*			
Anthropogenic sed	0.59	1.73	1.22 (± 0.36)
Original floodplain	0.38	0.77	0.61 (± 0.13)
*Fine sand (%)*			
Anthropogenic sed	0.31	0.85	0.59 (± 0.17)
Original floodplain	0.73	0.88	0.79 (± 0.04)
*Sand (%)*			
Anthropogenic sed	0.50	7.13	2.77 (± 1.62)
Original floodplain	2.03	5.52	3.96 (± 1.21)
*Mo*			
Anthropogenic sed	0.06	8.13	0.42 (± 1.22)
Original floodplain	0.07	0.63	0.19 (± 0.11)
*Cd*			
Anthropogenic sed	0.01	5.17	0.18 (± 0.79)
Original floodplain	0.00	0.12	0.03 (± 0.03)
*Sb*			
Anthropogenic sed	0.02	1.14	0.12 (± 0.23)
Original floodplain	0.01	2.49	0.26 (± 0.63)
*Se*			
Anthropogenic sed	0.38	2.07	1.57 (± 0.36)
Original floodplain	0.68	2.40	1.48 (± 0.43)
*Zn*			
Anthropogenic sed	0.81	105.84	39.69 (± 19.35)
Original floodplain	0.31	83.30	25.16 (± 15.92)
*W*			
Anthropogenic sed	0.01	0.12	0.04 (± 0.03)
Original floodplain	0.01	0.08	0.02 (± 0.01)
*Mg*			
Anthropogenic sed	297.14	3844	2077 (± 829.74)
Original floodplain	85.48	5208	1732 (± 1561)
*Al*			
Anthropogenic sed	2972	39,015	17,655 (± 7853)
Original floodplain	911.11	42,976	14,371 (± 12,816)
*Cr*			
Anthropogenic sed	5.36	39.10	22.13 (± 8.23)
Original floodplain	2.70	46.46	17.66 (± 13.07)
*Mn*			
Anthropogenic sed	37.37	480.15	189.99 (± 92.76)
Original floodplain	2.69	910.08	231.98 (± 280.34)
*Fe*			
Anthropogenic sed	3010	33,120	19,260 (± 8176)
Original floodplain	1396	30,351	12,245 (± 9164)
*Cu*			
Anthropogenic sed	1.98	332.78	27.09 (± 63.03)
Original floodplain	1.17	1261	106.38 (± 281.86)
*As*			
Anthropogenic sed	1.25	144	42.39 (± 28.66)
Original floodplain	0.83	13.40	4.24 (± 3.08)
*Pb*			
Anthropogenic sed	1.96	39.95	15.93 (± 8.07)
Original floodplain	1.10	103.19	16.48 (± 23.79)

**Fig. 2:**
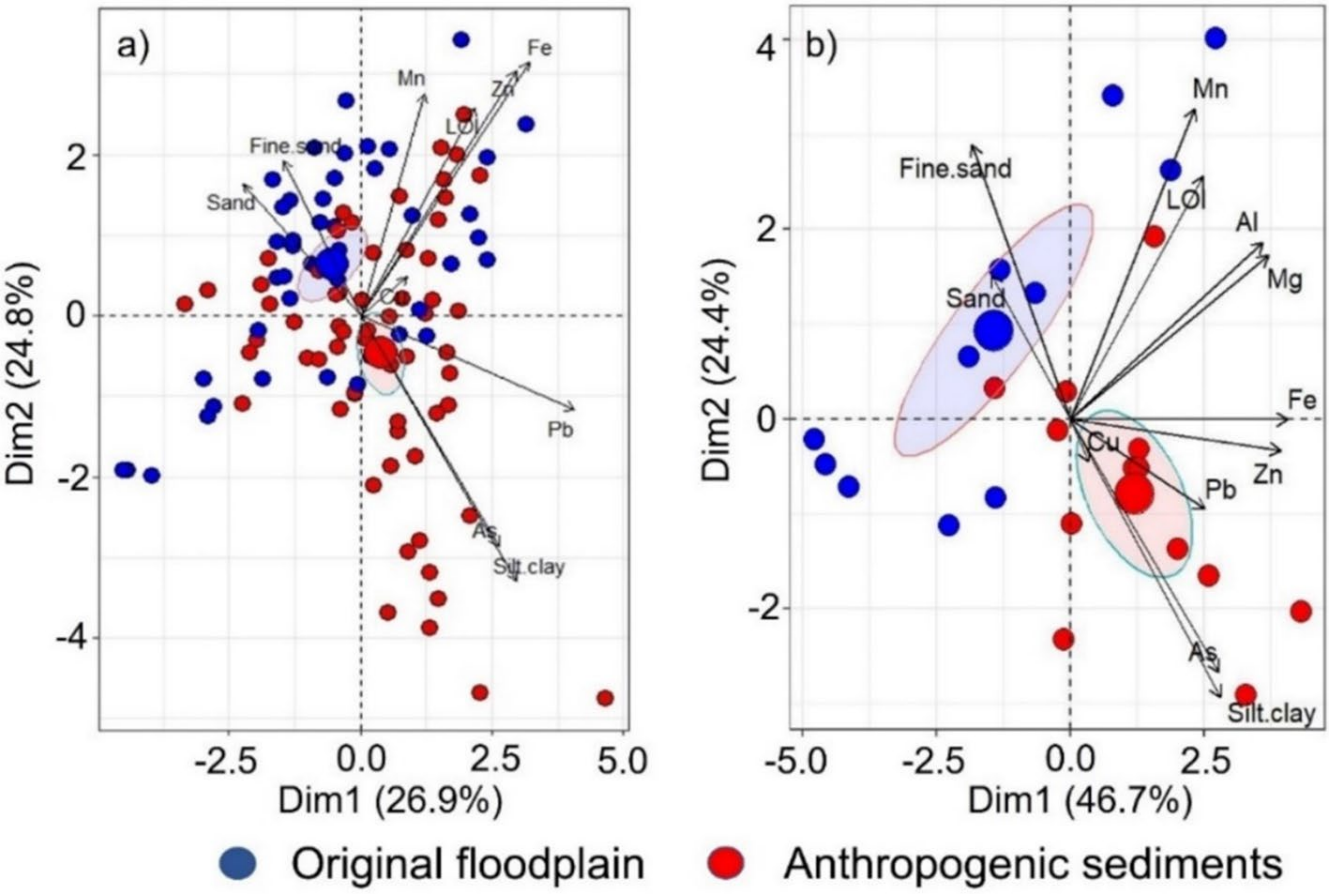
Principal component analysis diagrams for river-banks deposits of the Loddon River catchment. **a** Principal component analysis diagram for standardized Fe, Mn, Cu, Zn, As, Pb concentration measured by p-XRF, LOI (%), sand (%), fine sand (%), silt/clay (%) in anthropogenic and original floodplain sediments. **b** Principal component analysis diagram for standardized Fe, Mn, Mg, Al, Cr, V, Cu, As, Pb concentration measured by ICP-MS. LOI (%), sand (%), fine sand (%), silt/clay (%) in anthropogenic and original floodplain sediments from two river-bank deposits (Back Eddington and Bridgewater)

Overall, both methodologies highlighted a significant difference in arsenic concentration between anthropogenic and floodplain sediments. By contrast, ICP-MS analysis of the different size fractions revealed higher Cu and Pb concentrations in floodplain sediments, compared to the pseudo-total analysis performed by p-XRF during the field work. A principal component analysis (PCA) was applied to the data to visualize the relationships existing amongst the variables within the two groups (Fig. 2). The loadings for the PCA are reported in Supplementary Table S3. Figure 2a reports the PCA results from analysis performed with p-XRF data for all the investigated sites. The two principal components extracted explained approximately 54% (PC1: 29.82%; PC2: 23.47%) of the variability across all samples. Arsenic and Pb were strongly associated with the first and second components, respectively. The analysis of similarities (ANOSIM) calculated a sample statistic (R) of 0.126 and a statistically significant difference between the anthropogenic sediments and the original floodplain sediments (*p* = 0.001). SIMPER analysis indicated a degree of similarities of 67.5%, for anthropogenic and floodplain sediments, respectively.

Standardized data from ICP-MS analysis from Back Eddington and Bridgewater sites were also analysed by PCA to further assess the association between variables within the two sediment types (anthropogenic and floodplain sediments) (Fig. 2b). The loadings for the PCA are reported in Supplementary Table S4. Approximately 72% of the data variability was explained by two principal components (PC1: 46.7%; PC2: 24.4%). Anthropogenic sediments were significantly different from the original floodplain (ANOSIM: sample statistic R of 0.188; *p* < 0.001), with clear separation along the PC2 axis. This separation was mainly related to the texture of the deposits and metals such as Pb and As (both relatively elevated in the anthropogenic sediment). Sediment variation along the PC1 axis was driven by other metals such as Fe, Zn, Mg and Al. Overall, the PCA results from ICP-MS data for the two sites (Fig. 2b) were similar to the p-XRF results from all sites (Fig. 2a) and separated anthropogenic and floodplain sediments in terms of silt/clay content and As level. As observed for the p-XRF results, the other metals were not strongly associated to differences between the two layers.

### Arsenic-spatial/depth distribution

**Fig. 3:**
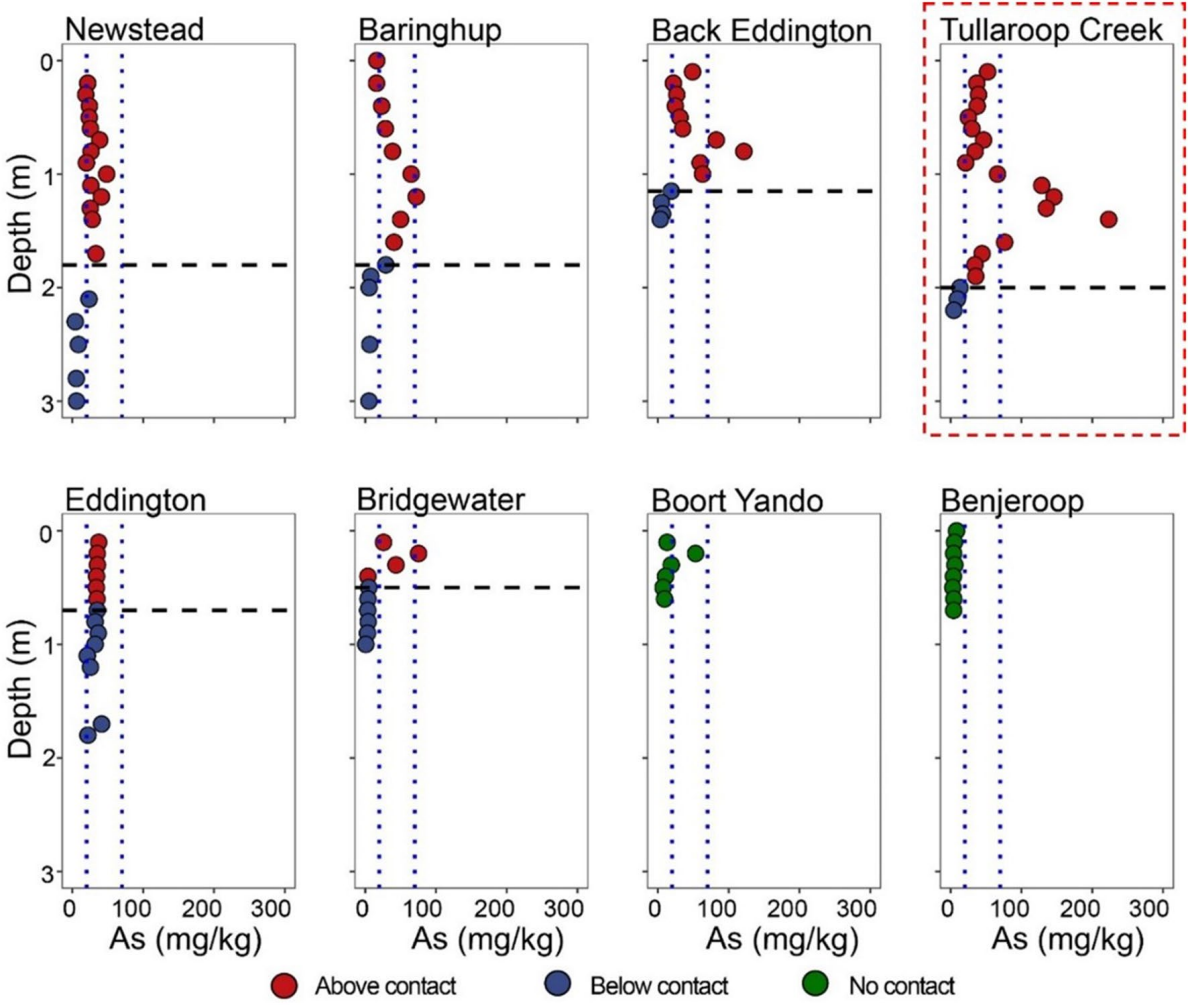
Arsenic profiles for the eight river-bank deposits along the Loddon River and Tullaroop tributary. Profiles extend through the anthropogenic sediments through to original (relic) floodplain (boundary marked by horizontal dotted line). Arsenic measurements by p-XRF. Dotted red box = tributary Tullaroop Creek. Vertical dotted lines represent the low level (20 ppm) and high level (70 ppm) of the interim sediment quality guidelines (ISQG) for Australia and New Zealand. For locations of sites refer to Fig. 1

Of all metals and metalloids analysed, As was found to be most elevated in anthropogenic sediments relative to the original floodplain deposits. The majority of upstream profiles (sites 1, 2, 3 and 4) had higher levels of As within the anthropogenic sediments, in the form of a submerged “plume” (Fig. 3). This maximum occurred at 1 m ± 0.4 depth for three of the investigated sites (Baringhup, Back Eddington, Tullaroop Creek). The As plume became more restricted within the profile moving downstream, commensurate with the decreasing depth of the anthropogenic layer. Arsenic concentration increased from site 1 to site 4, before decreasing again (Supplementary Figure S8). All the sites investigated, except for Benjeroop, contained As concentrations in the overlying anthropogenic deposits that exceeded the ISQG low trigger value (20 ppm). Baringhup, Back Eddington, Bridgewater and Tullaroop Creek also had samples exceeding the As ISQG high trigger value (70 ppm), at depths of: 1.2 (Baringhup), 0.7–0.8 (Back Eddington), 0.2 (Bridgewater) and 1.1–1.6 (Tullaroop Ck) meters.

**Fig. 4:**
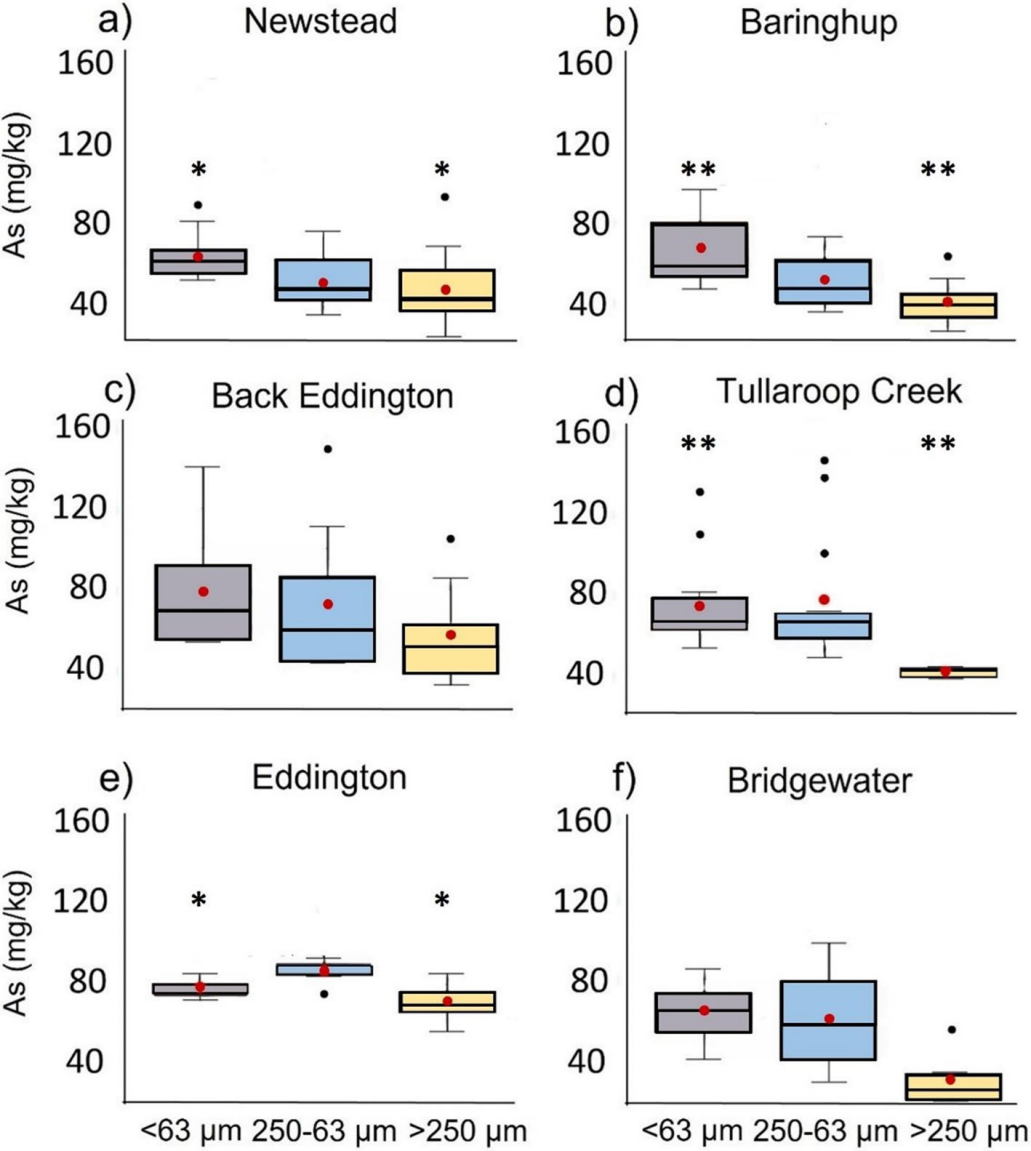
Box and whisker plot of arsenic concentrations (mg/kg) in anthropogenic sediments from p-XRF measurements for the > 250 μm, 250–63 μm and < 63 μm size fractions obtained for the eight river-bank deposits. The box indicates the 1st and 3rd quartiles, and the dark line indicates the median. The mean is indicated with red dots. Significant differences between groups are indicated with *(< 0.05) and **(< 0.01)

A negative relationship was observed between grain-size and the arsenic concentration for the anthropogenic sediments at 4 of the 6 sites, with a significant difference (< 0.01) between the clay/silt fraction (< 63 µm) and the sand fraction (> 250 µm) (Fig. 4). Of the two most downstream sites, Boort Yando showed elevated As levels in the sand fraction (> 250 µm) (Figure S9). Overall, this preferential association of As with finer sizes of sediments (250–63 µm and < 63 µm) reflects the higher surface area of silt and clay compared to larger-size sand particles. The relationship with grain size and vertical trends exhibited by selected metals obtained by p-XRF and ICP-MS analysis are reported in SI (Supplementary Information Figures S9 to S23).

### Arsenic clustering around Victoria

The map in Figure S24 shows the kernel density estimation for arsenic from surface samples from the GeoVic database. The results indicate that multiple arsenic hotspots are present across Victoria. The VicMine data includes > 10.000 of sites across the state, with analysis from surface soil samples. Kernel density surfaces are reported with increasing value corresponding to the increasing density of high levels of As in surface samples within the surface. The kernel density surface highlighted in blue in the right panel of Figure S24 is characterized 1200 points, of which only 20 with As concentration higher than 300 ppm. By comparison, of just 8 sites investigated in this study, at the Tullaroop Creek site, As concentration higher than 300 ppm were detected at a depth of 1.4–1.5 m.

## Discussion

Previous work has documented the crucial role anthropogenic activities have played in As contamination of the Australian environment, particularly mining (Smith et al., 2003, Medunić et al., 2020). Arsenic concentrations in this study ranged from 16 to up to 312 mg/kg. Arsenic concentrations were compared to the trigger values (ISQG) for Australia and New Zealand, that is the low (20 ppm) and high (70 ppm) thresholds, above which adverse biological effects are expected to occur (Simpson et al., 2013). From chemical analyses, it was shown that anthropogenic sediments in the Loddon River floodplain are contaminated with As, often beyond the high thresholds of 70 ppm. These As values are also higher than estimates made by a review specifically addressed for freshwater sediments, that indicated arsenic threshold values of 9.79 mg/kg (TEC: Threshold Effect Concentration) and 33 mg/kg (PEC: Probable Effect Concentration) for the absence or presence of harmful effects, respectively (MacDonald et al., 2000).

Previous studies looking at Victorian goldfield mine wastes recorded arsenic concentrations in battery and calcine sands ranging from 265 to up to 15,000 mg/kg (Martin et al., 2016). High As values up to 390 mg/kg were also found near mine tailing deposits in the Ballarat area (Sultan, 2007), as high as 900 mg/kg As in soils near the Stawell Gold Mine area (Noble et al., 2010) and up to 185 mg/kg at Maldon mine sites (Abraham et al., 2018). Similar As contamination of soils associated with historic gold mining has been reported in New Zealand (Craw et al., 2000; Haffert et al., 2010; Kerr & Craw, 2021) and California (Alpers, 2017; Savage et al., 2000). In this study, anthropogenic sediments along the Loddon River in Victoria have been shown to contain higher concentrations of contaminants (mainly arsenic) and a different textural composition than the underlying original floodplain deposits. More importantly, it has been found that this is not a localized phenomenon but is evident for more than 60 kms downstream from the sites of contamination. The As maxima at around 1 m depth for three of the upstream sites (Baringhup, Back Eddington, Tullaroop Creek; Fig. 3) and the reduction in the arsenic maximum proceeding down the catchments (Supplementary Figure S7), suggests that As was accumulated on floodplains throughout overbank flows of mine tailings during the historical gold rush and reworked over time (Ciszewski & Grygar, 2016; James, 2013; Lawrence et al., 2016; Parker et al., 2022).

The apparent formation of a submerged plume that disappears at the two most downstream sites (Boort Yando, Benjeroop) may be due to dilution processes, as a result of the input into the system of uncontaminated sediments through gully erosion or changing levels of As contamination during the different stages of mining (Ciszewski & Grygar, 2016; Lecce & Pavlowsky, 2014; Lewin & Macklin, 1987; Macklin et al., 2006), or simply because of the narrowing of the plume moving downstream, due to the flat terrain of the Loddon floodplain compared to the more confined parts of the upstream river channel. Furthermore, arsenic may be temporarily trapped during secondary diagenesis, by co-precipitation and sorption on oxides and hydroxides (Hudson-Edwards et al., 1999; Sowers et al., 2017; Tokoro et al., 2020). This process could explain the elevated As values in the lower part of the profiles analysed, probably operating during the early stage of accumulation of these sediments on the Loddon River floodplains. The presence of the As-plume at depth and the numerous arsenic hotspots throughout Victoria, as depicted in Figure S24, raises significant environmental and potential human health concerns. Elevated levels of arsenic detected at depth, as indicated in this study, pose challenges for detection through surface sampling alone. Therefore, further investigations are necessary to assess the presence of higher arsenic concentrations in deeper layers, particularly in the hotspots identified from the VICMine data. Despite the arsenic plume being detected at depths of at least 1.5 m, changing environmental conditions could potentially mobilize As, leading to its migration towards surface layers. This raises concerns about potential environmental effects and uptake by plants and subsequent entry to the terrestrial and aquatic food webs. Moreover, the As plume may also pose health risks to humans via consumption of crops potentially grown in contaminated regions. Previous research, conducted by Pearce et al., (2012) have reported a rise in the risk of cancer linked to elevated soil arsenic levels in historical mining regions in Victoria, while Martin et al., (2013) found correlations between soil As concentrations and As in the toenail clippings of children living in contaminated areas (Martin et al., 2013; Pearce et al., 2012). Thus, it is imperative to institute rigorous monitoring of exposure pathways and implement comprehensive environmental screening protocols that take into account the potential presence of As plumes at depth and to evaluate the chronic and potentially acute effects of arsenic exposure.

**Fig. 5:**
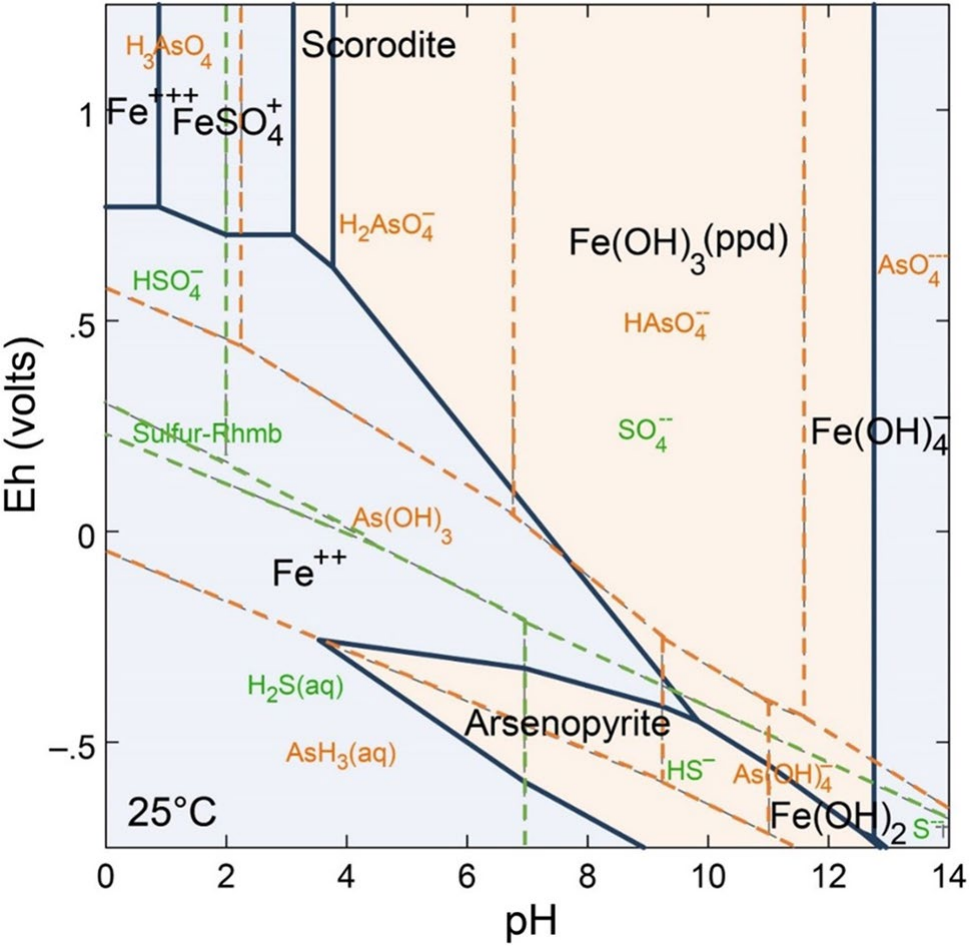
Pourbaix diagram (Eh–pH) showing the stability fields for aqueous As species, Fe and As phases (activities: Fe = 10^−3^ M; As, S = 10^−6^ M; HCO_3_ = 10^−4^ M); from Craw and Bowell (2014)

Mean arsenic values in the anthropogenic sediments from the Loddon River catchment exceeded both the Australian and Victorian mean soil background concentrations for As of 3 and 5 mg/kg, respectively (de Caritat & Cooper, 2011, 2016). Arsenic concentrations higher than background averages in mining areas are often related to the weathering of arsenopyrite (FeAsS) (Phillips & Hughes, 1996; Rae, 2001; Sultan, 2007; Wen et al., 2022). Arsenic mobility is therefore, strongly controlled by arsenopyrite geochemistry (Craw & Bowell, 2014). Arsenopyrite is stable under reducing conditions, but once exposed to the atmosphere, in carbonate-poor systems the accelerated oxidation enhanced by the mining processes (e.g., crushing and grinding) leads to the surface dissolution of arsenopyrite with the subsequent formation of As (V) and As (III) acids and sulfur species (Corkhill & Vaughan, 2009; Craw & Bowell, 2014). The process of arsenopyrite oxidation may be also catalyzed by bacteria (Dove & Rimstidt, 1985; Kawa et al., 2019). By contrast, under oxidizing conditions arsenic is either in the form of scorodite or soluble arsenate species depending on pH (Fig. 5). Arsenic temporarily incorporated into scorodite minerals (FeAsO_4_*2H_2_O) may dissolve incongruently (pH > 4) according to reactions 1 (Dove & Rimstidt, 1985; Harvey et al., 2006):

1 $${\text{FeAs}}{{\text{O}}_{4}}*{{\text{H}}_{2}}{\text{O}} + {{\text{H}}_{2}}{\text{O}} = {{\text{H}}_{2}}{\text{AsO}}_4^- + {\text{Fe}}{({\text{OH}})_{3}}({\text{s}}) + {{\text{H}}^+ }$$

The dissolution of scorodite liberates iron hydroxides and arsenate oxyanions (Dove & Rimstidt, 1985; Harvey et al., 2006), released into the environment along with mine tailings and/or as dissolved species in wastewaters. However, given the high affinity of As for Fe oxyhydroxides, as incongruent dissolution proceed, As adsorption to iron oxyhydroxides may affect its concentration in solution (Harvey et al., 2006; Zhang et al., 2019).

The presence of high concentrations of Pb and Cu may derive by the presence in the area of metal sulfides commonly associated with gold mineralisation in Victoria, such as galena (PbS) and chalcopyrite (CuFeS_2_) (Phillips & Hughes, 1996; Sultan, 2007). Whether Pb and Cu occur as discrete mineral phases is unknown at these sites. High Pb and Cu concentrations detected within some of the floodplain deposits (sink site) may indicate that a different post-depositional evolution relative to arsenic may have occurred, since levels high in Pb and Cu are below the contact between anthropogenic sediments and floodplain deposits. This different vertical pattern (Fig. 3, S22 and S23) suggests that vertical migration of arsenic is negligible relative to those of Cu and Pb. The apparent migration of some metals (Cu and Pb) into the floodplain relative to As could be explained by the different behaviour of oxyanions compared to metal cations, that may have partitioned the metals into relatively more mobile solid phases compared to arsenic. While arsenic tends to adsorb strongly onto solid phases such as iron oxides, copper and Pb form soluble complexes, enhancing their mobility in the soil environment.

In Central Victoria, common methodologies adopted for the extraction of gold were puddlers and stamp batteries. These practices released into the waterways sediments ranging in composition from clay to sand (Davies et al., 2018a, 2018b). This process of accelerated sediment delivery from the upstream areas of the catchment to the downstream reaches resulted in an aggradation often so critical in some areas that it lead to the filling of the whole channel, as in the case of Tullaroop Creek (Grove et al., 2019). These sludge sediments, mobilised in less than half a century, accounted for a volume of around 80 × 10^6^ m^3^ just for the Loddon River catchment (Davies et al., 2018a, 2018b). The same authors concluded that between 1859 and 1891, a total volume of around 500 million cubic metres of mining sediments had been dispersed along Victorian catchments. Our results support the findings by some authors that highlighted how floodplains in Victoria River catchments are deeply affected by historical gold mining (Davies et al., 2018a, 2018b; Lawrence et al., 2016). It has been recognised that floodplain systems may contain large amounts of potentially contaminated sediments, such as mine tailings produced during historical gold mining (Macklin et al., 2023). Accordingly, it has been estimated that around 90% of the sediments discharged by more than a century of mining in Victoria, are now part of the sediments on floodplains of the Loddon River and its tributaries (Abernethy et al., 2004). Grove et al., 2019 compared estimates of changes associated with land clearing to sludge volumes, modelled by CSIRO using the sediment budget network (SEDNET) model (Prosser et al., 2001) and found that sediment loads associated with mining were of orders of magnitude higher than natural levels (Grove et al., 2019).

Mine tailings range in textural composition from gravel to silt, depending on the methodology used to extract the ore and on the deposit being mined (Lottermoser, 2010). In a complex scenario such as the Loddon catchment during the gold rush, different technologies were used to extract gold both from alluvial and quartz veins deposits (Davies et al., 2018a, 2018b; Grove et al., 2019). Therefore, it is not unexpected to find that the anthropogenic sediments in this study were characterized by different size fractions. The highly variable particle sizes and poor sorting (Figure S3), the presence of particles with sharp edge morphology (similar to those observed in tailing heaps, Figure S4, SI), coupled with the high As values found in this study are all indicators that these post-European deposits contain mine tailings discharged from mine sites.

Gold mining in Victoria during the last century has left an enduring legacy in the landscape. Starting from the more visible artefacts, like remnants of infrastructure for water supply, sluiced gullies, dredge ponds, sludge channels, dredges and the different stratigraphy to the less obvious impact that their waste product (i.e., sludge) have had on the downstream floodplains (Lawrence et al., 2016, 2021). These anthropogenic sediments contain metals in concentrations that are potentially hazardous for the environment. In Victoria, thousands of sites across the state are characterized by surficial arsenic contamination (Figure S24). This work indicated that subsurface concentration of As could be substantially higher than surface samples. As a consequence, other catchments draining areas where historical gold mining occurred may be potentially have higher As concentrations at depth.

## Conclusion

The historical gold rush during the period 1851–1914 greatly affected the Loddon River catchment, like many other river systems in Victoria. The adverse impact of mining has resulted primarily in the presence of high concentrations of harmful contaminants (i.e., arsenic) in the sub-surface sediments, up to 60 kms down the catchment. The presence of sediments containing high levels of As is likely a consequence of the poorly regulated delivery of enormous volumes of wastes into the waterways by miners using relatively primitive processing methods.

Secondly, the texture and composition of these anthropogenic sediments differs compared to the underlying original floodplain deposits, as evidenced by particle characteristics, such as texture, PSD features, particle morphology and thereby their mechanical behaviour (i.e., competence, transport, deposition). All these attributes have an important influence on the changes of the river floodplain. Four sites were found to have arsenic concentrations in anthropogenic sediments higher than the SQGV-high of 70 ppm set by ANZECC (Simpson et al., 2013), thereby potentially posing a significant risk to human health and the environment.

Dynamic conditions such as changes in wetting and drying cycles, typical of natural environments, may remobilized contaminants from these fluvial sediments. Therefore, Victorian riverine floodplains, such as those in the Loddon River (this study), are still likely adversely impacted by the historical gold rush, well after more than a century since intensive mining activity. In addition, a large proportion of these As-contaminated sediments are upstream of major reservoirs (e.g., Cairn Curran, Laanecoorie). Hence, further studies are required to estimate the chemical and physical forms of the As within these sediments to assess potential mobility, bioavailability and toxicity. Future studies should determine: (a) the mineral phases of arsenic present in these anthropogenic sediments; (b) the speciation of As and its distribution in the different solid sediment phases (labile, sorbed, mineral bound) in order to establish its potential mobility, toxicity and bioavailability, since total metal contents in sediments provide little information regarding the potential mobility, bioavailability and toxicity (Foster & Kim, 2014; Wenzel et al., 2001).

### Supplementary Information

Supplementary file 1.

## Data Availability

The authors declare that the data supporting the findings of this study are available within the paper and its Supplementary Information files. Should any raw data files be needed in another format they are available from the corresponding author upon reasonable request. Source data are provided with this paper.
