# Supplementary material for: Legacy effects of historical gold mining on floodplains of an Australian river
Source: Environ Geochem Health. 2024 Jun 13;46(7):247. doi: 10.1007/s10653-024-02003-5 (PMC11176104; doi:10.1007/s10653-024-02003-5)
Supplement: Supplementary file 1 [file 10653_2024_2003_MOESM1_ESM.docx]

**_Supplementary Information_**

Legacy effects of historical gold mining on floodplains of an Australian river

Francesco Colombi¹*, Aleicia Holland¹, Darren Baldwin²,³, Susan Lawrence⁴, Peter Davies⁴, Ian Rutherfurd⁵, James Grove⁵, Jodi Turnbull⁴, Mark Macklin⁶, Greg Hil⁴ & Ewen Silvester¹

¹Department of Environment and Genetics, School of Agriculture, Biomedicine and Environment, La Trobe University, Albury/Wodonga Campus, Wodonga, VIC 3690, Australia

²School of Agricultural, Environmental and Veterinary Sciences, Charles Sturt University, Thurgoona, NSW, 2640, Australia

³River and Wetlands, Victoria, Australia

⁴Department of Archaeology and History, La Trobe University, Bundoora, Victoria, 3086, Australia

⁵School of Geography, Faculty of Earth and Atmospheric Science, University of Melbourne, 22 Bouverie Street, Melbourne, Victoria, 3001, Australia

⁶School of Geography & Lincoln Centre for Water and Planetary Health, College of Science, University of Lincoln, Lincoln, Lincolnshire LN6 TS, United Kingdom

*Corresponding author: Francesco Colombi, Department of Environment and Genetics, Centre for Freshwater Ecosystems, School of Agriculture, Biomedicine and Environment, La Trobe University, Albury/Wodonga Campus, Wodonga, VIC 3690, Australia

Email: 19574689@students.ltu.edu.au; francesco.colombi89@gmail.com

Phone number: +61 490208185

Summary of the Supporting information

• Number of pages: 33 (including this page)

• Number of figures: 24

• Number of tables: 4

**Supporting Information contains**:

**Figure S1**. Photographs of the river-bank deposits investigated along the catchment of the Loddon river

**Figure S2**. Photograph of anthropogenic sediments overlying original floodplain deposits

**Figure S3**. Ternary diagrams showing the grain size distribution for each riverbank profile

**Figure S4**. Photographs of sand particles under microscope

**Figure S5**. Particle-size distribution (PSD) curves for the eight river-bank deposits

**Figure S6**. Loss on ignition (LOI) for the eight river-bank deposits along the Loddon River

**Figure S7**. Arsenic (mg/kg) versus depth (m) for the eight river-bank deposits

**Figure S8**. Arsenic (mg/kg) versus distance (km)

**Figure S9**. Box and whisker plots of arsenic concentrations (mg/kg) for the >250μm, 250-63μm and <63μm size fractions (p-XRF)

**Figure S10-S13**. Box and whisker plots of metals concentrations (mg/kg) for the >250μm, 250-63μm and <63μm size fractions (ICP-MS)

**Figure S14-S21**. Metals (mg/kg, g/kg) versus depth (m) for the eight river-bank deposits (p-XRF)

**Figure S22-S23**. Metals (mg/kg, g/kg) versus depth (m) for the two river-bank deposits (ICP-MS)

**Figure S24**. The Kernel Density map for arsenic clustering around Victoria, Australia

**Table S1**. Concentrations (mg/kg) of metals (p-XRF) in river-bank deposits of the Loddon River

**Table S2**. Concentrations (mg/kg) of metals (ICP-MS) in river-bank deposits of the Loddon River

**Table S3**. Principal component analysis of all river-bank deposits

**Table S4**. Principal component analysis of two river-bank deposits

**Section 1** Other Metals

At Back Eddington and Bridgewater sites, two samples analysed by ICP-MS showed anomalously high Pb and Cu concentrations in floodplain sediments. Specifically, Back Eddington displayed high Cu values in the finest (<63µm) and coarser (>250µm) fractions in both the anthropogenic and floodplain sediments (Figures S10-S11). Bridgewater showed high Pb and Cu concentrations in floodplain sediments in the coarser fractions (>250µm and 250-63µm) (Figures S12-S13). Copper and Pb concentrations exceeded the ISQG low trigger value of 65 ppm and 50 ppm, respectively. High metal concentration in the underlying floodplain sediments were found at depths of 1.3 and 0.7 meters for Back Eddington and Bridgewater, respectively. Box and whisker plots showed again a negative trend between grain-size fractions and the metal concentration of the deposits (Figures S10 to S13), confirming that grain-size is one of the essential factors influencing the content of metals in soils.

Depth profiles from p-XRF data showed similar distribution of metals such as Fe, Mn and Cr between the anthropogenic sediments and the underlying original floodplain (Supplementary Figures S14 to S21). Depth profiles of the abovementioned metals in different size fractions analysed by ICP-MS also displayed consistent patterns with the p-XRF data (Figures S22-S23). Back Eddington displays a peak in Fe in the 250-63µm fraction and in Pb in the coarser fraction (<>250µm). Both peaks occur within the anthropogenic sediments. A sample from this site was also characterized by a high Cu content in the finest and coarser fractions. By comparison, Bridgewater showed an increase in most metals, except for Cu.


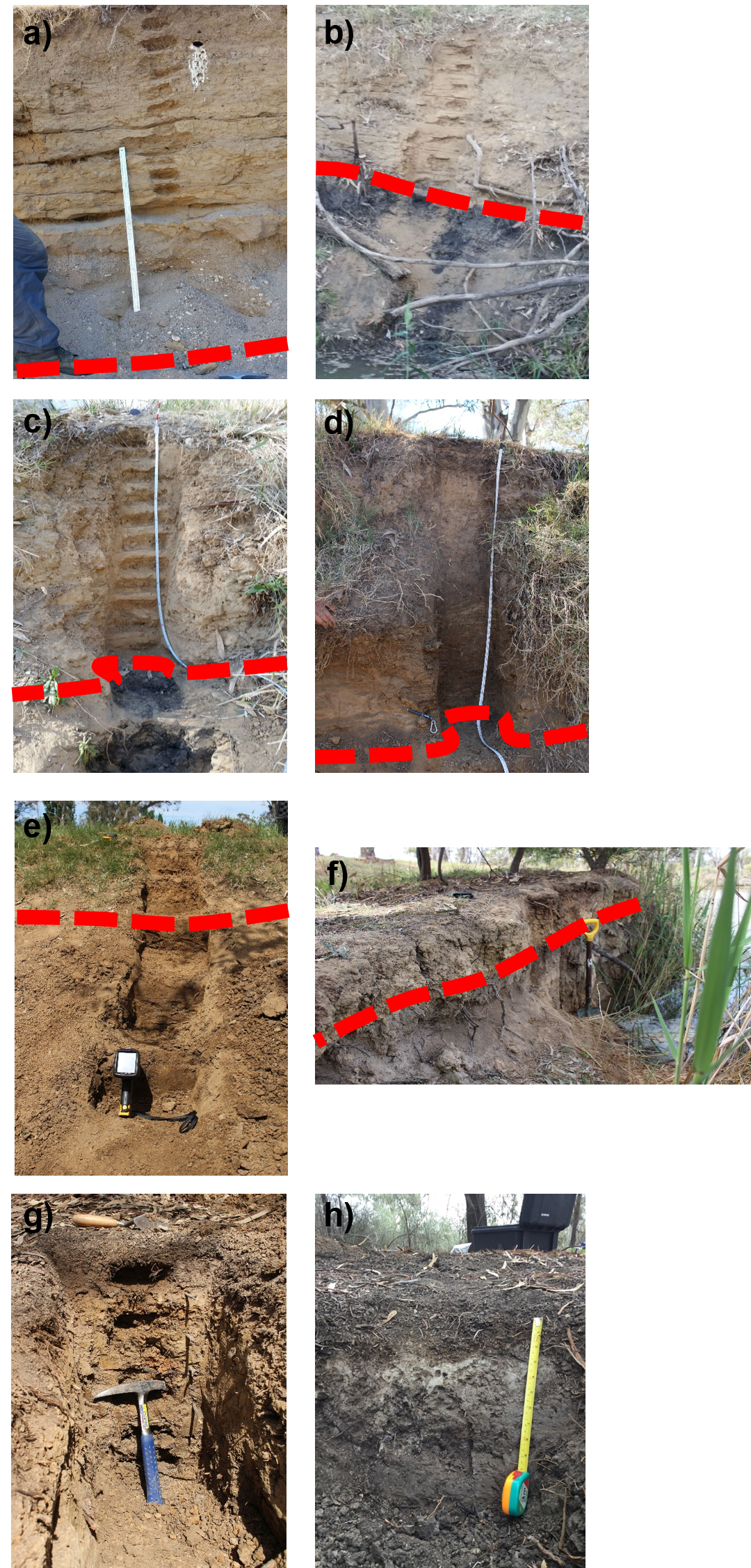


**Fig. S1**: Photographs of the eight river-bank deposits analysed along the catchment of the Loddon River: a) Newstead; b) Baringhup; c) Back Eddington; d) Tullaroop Creek; e) Eddington; f) Bridgewater; g) Boort Yando; h) Benjeroop. The photos are disposed in spatial order, from the upstream site (Newstead) to the downstream site (Benjeroop). The red dotted line represents the contact between the anthropogenic sediments on top of the original (pre-existing) floodplain sediments. In the two most downstream sites, Boort Yando (g) and Benjeroop (h), the overlying anthropogenic sediments are no longer detectable. The thickness of the anthropogenic sediments is reported: Newstead-2.1 m; Baringhup-1.8 m; Back Eddington- 1.15 m; Tullaroop Creek-1.9 m; Eddington-0.7 m; Bridgewater-0.4 m


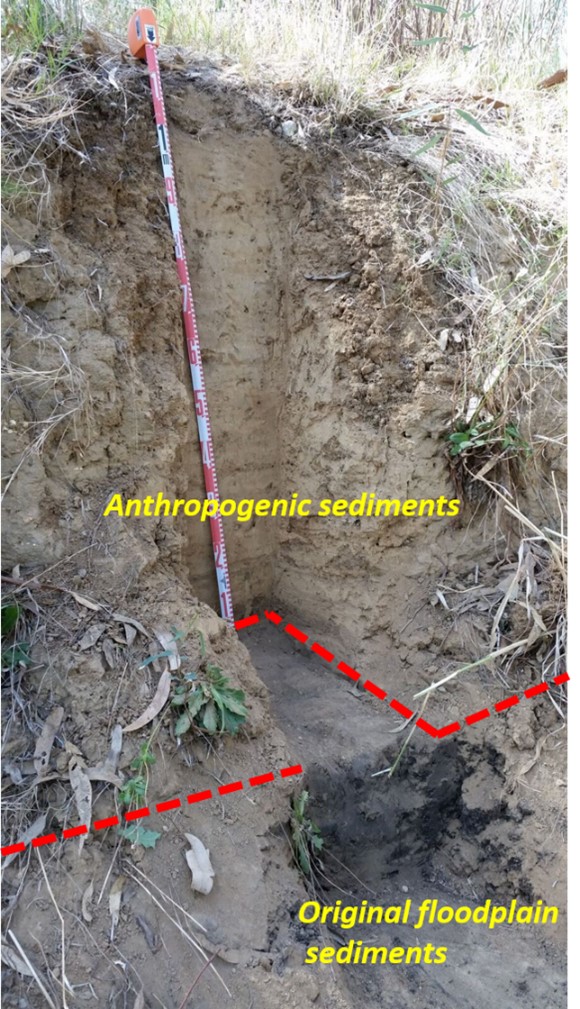


**Fig. S2**: Photograph of anthropogenic sediments overlying original floodplain deposits (contact marked by the red line; Baringhup, Loddon River)


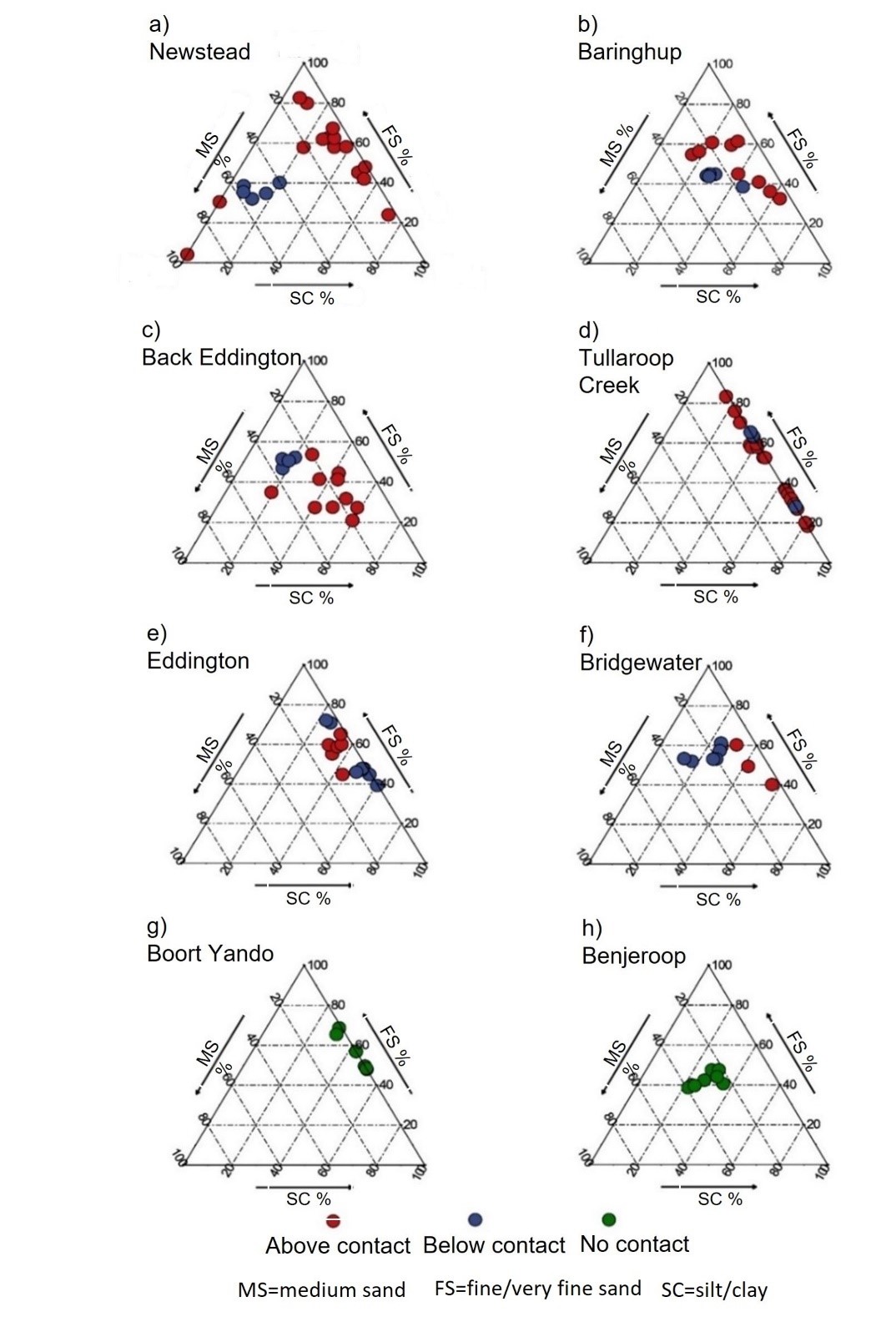


**Fig. S3**: Ternary diagrams showing the grain size distribution for each of the investigated profile (Shepard 1954)


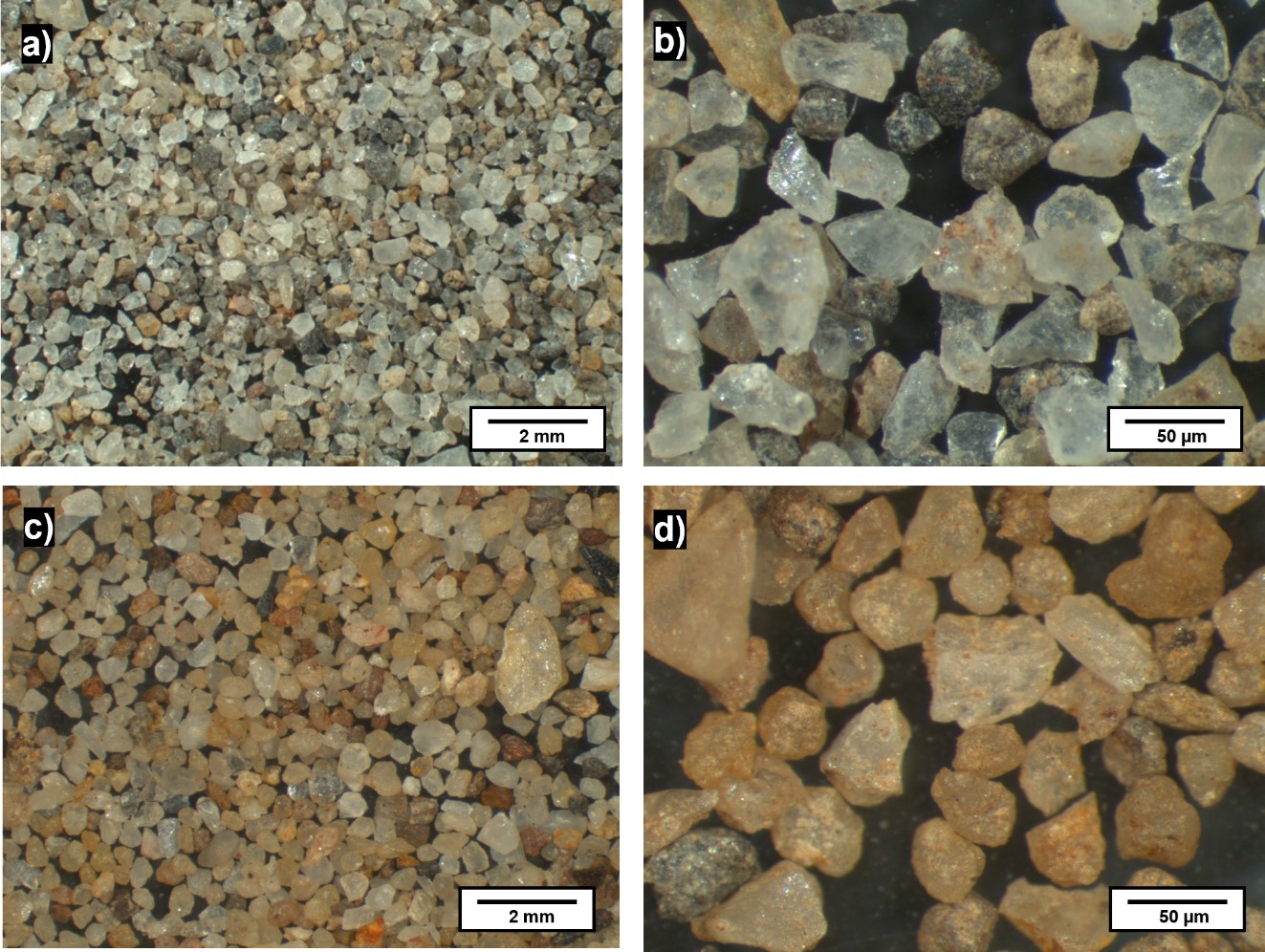


**Fig. S4**: Sand particles under the microscope: a)-b) tailing heaps from North British Mine, Victoria, Australia; c)-d) re-deposited anthropogenic sediments from the Newstead site


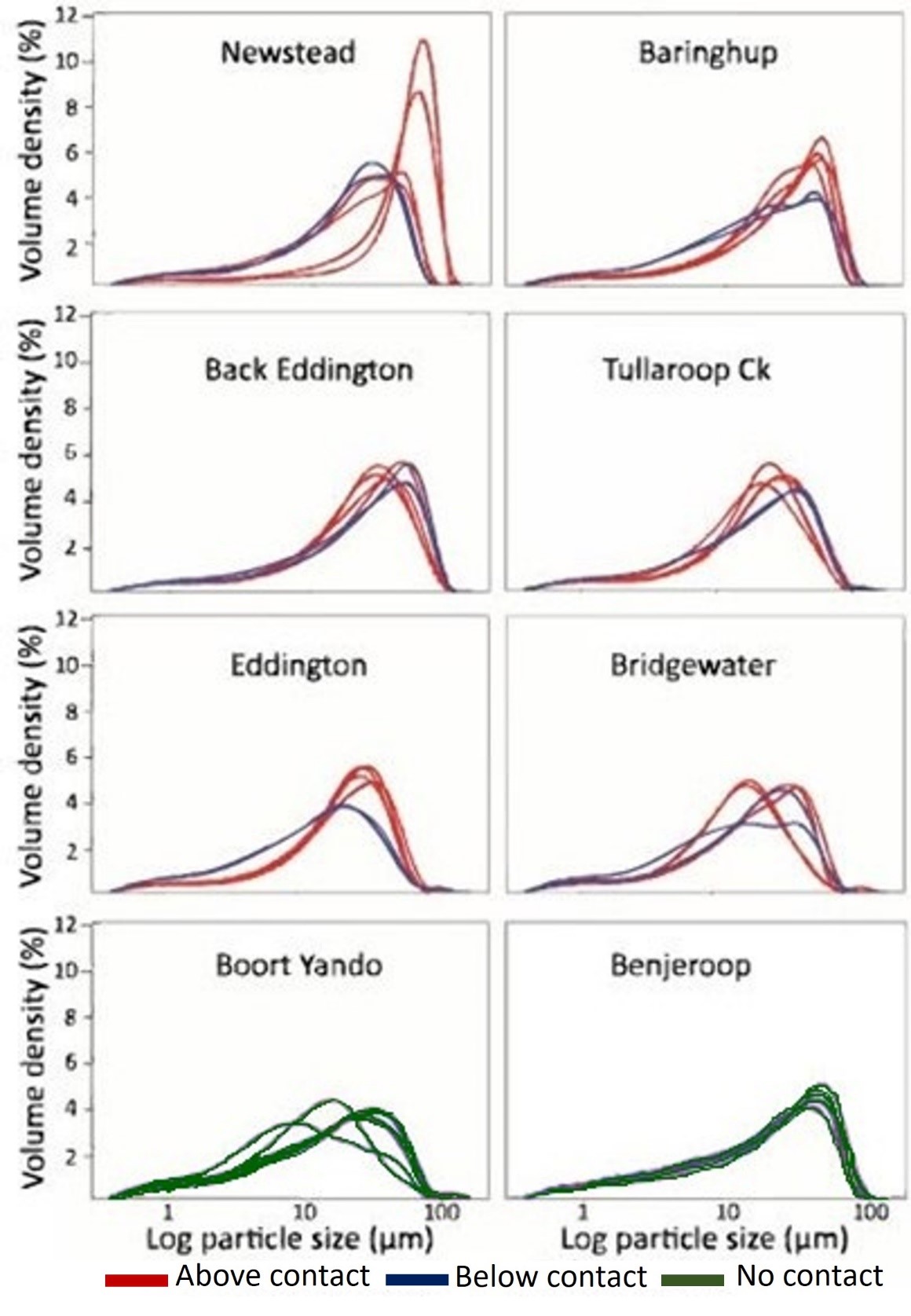


**Fig. S5**: Particle-size distribution (PSD) curves for the eight river-bank deposits along the Loddon River. Red distribution curves represent the anthropogenic sediment samples; blue distribution curves represent the original floodplain sediments


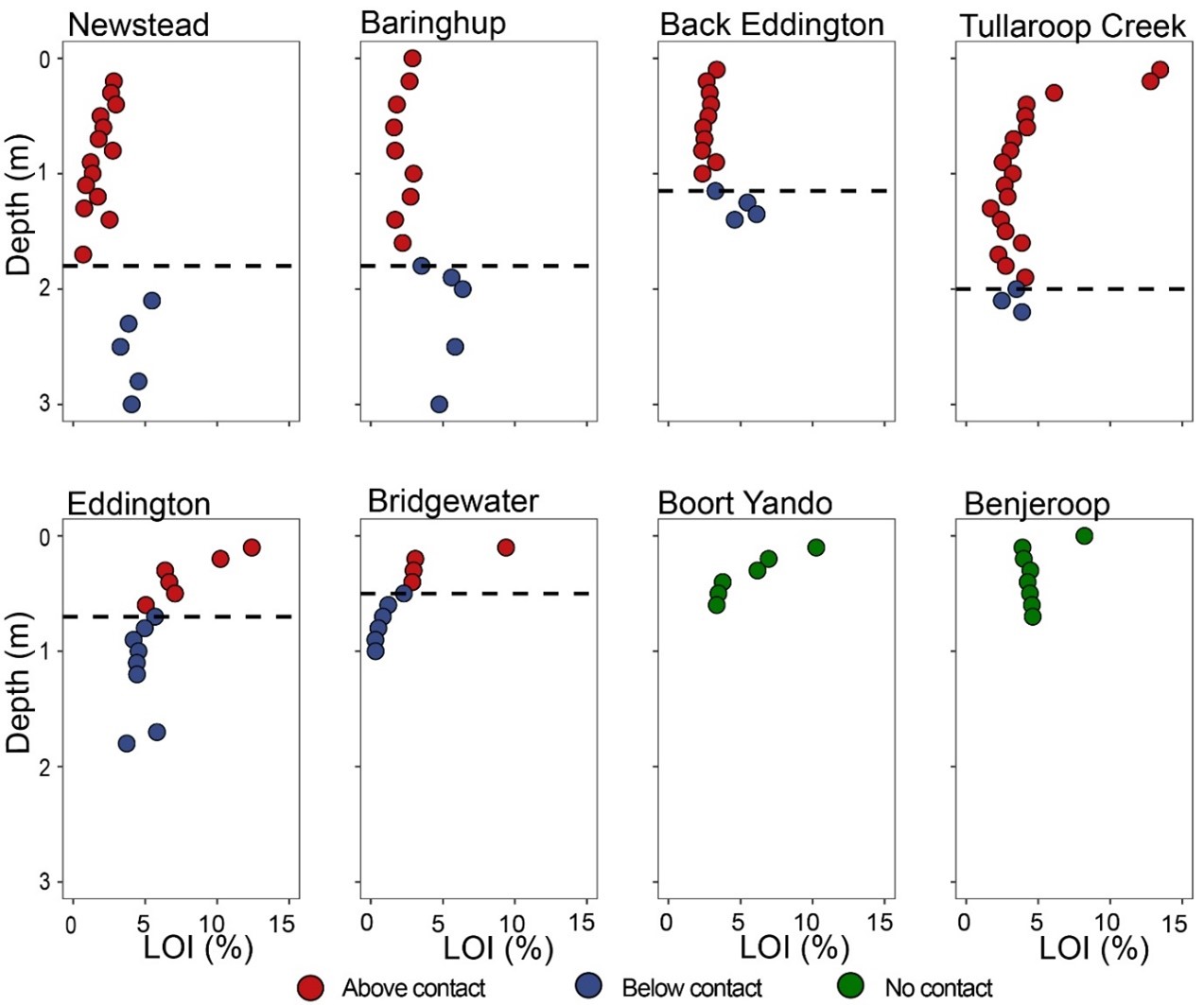


**Fig. S6**: Loss on ignition (LOI) for the eight river-bank deposits along the Loddon River. Profiles extend through the overlying anthropogenic sediments through to original (relic) floodplain (boundary marked by horizontal dotted line) LOI is a poxy measure of total organic carbon


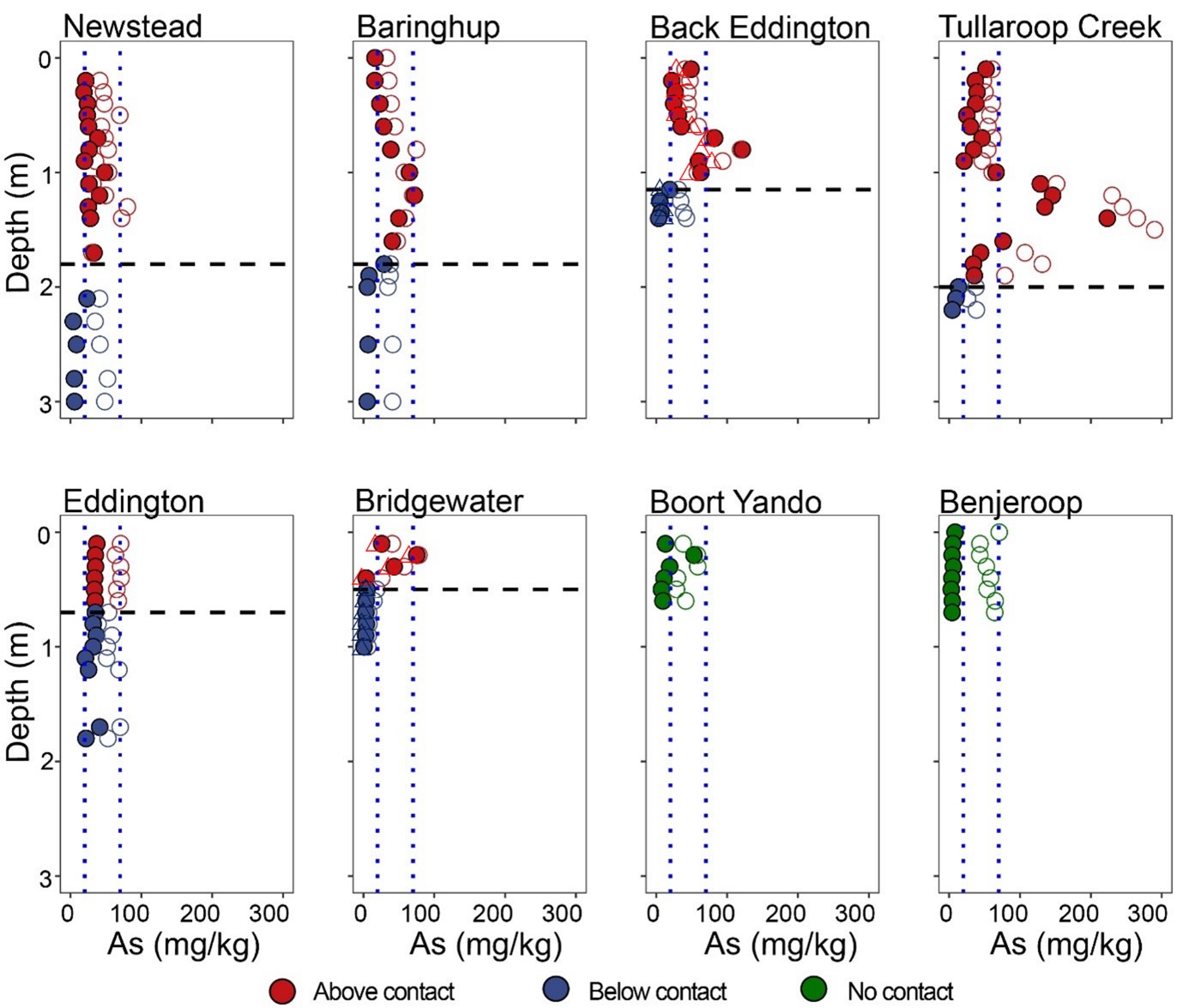


**Fig. S7**: Arsenic profiles for the eight river-bank deposits along the Loddon River. Profiles extend through the anthropic sediments through to original (relic) floodplain (boundary marked by horizontal dotted line). Full circles = total arsenic measured via p-XRF; open circles = total arsenic measured via graphite furnace atomic absorption spectroscopy (GF-AAS); open triangles = total arsenic measured via ICP-MS (for two profiles only). Dotted red box = tributary Tullaroop Creek. Blue vertical dotted lines represent the low level (20ppm) and high level (70ppm) of interim sediment quality guidelines (ISQG) for Australia and New Zealand. For profiles name refer to Figure 1


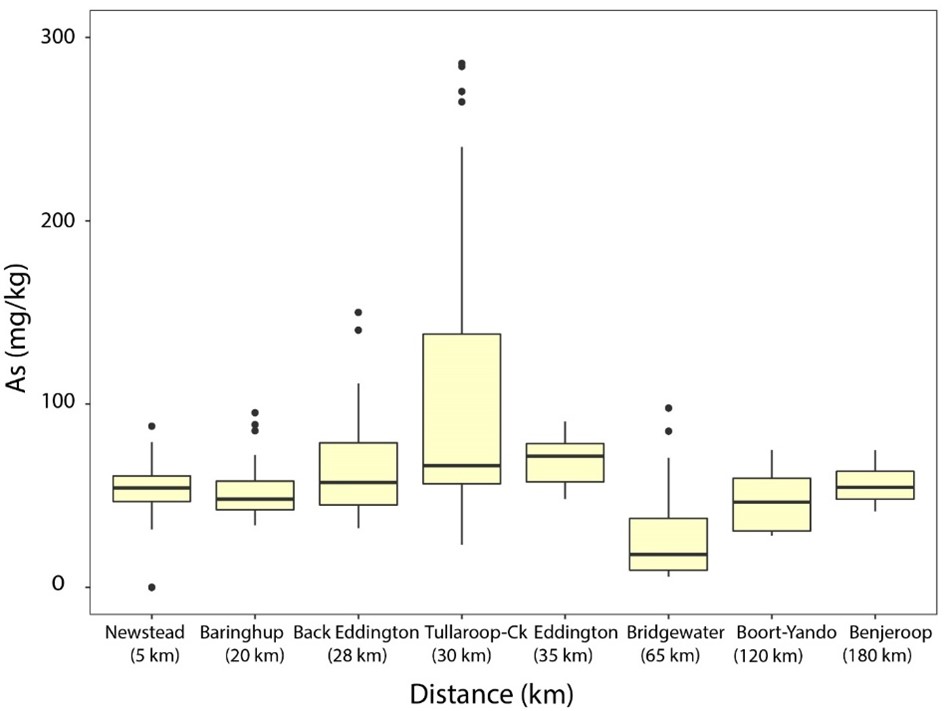


**Fig. S8**: Box plots of arsenic concentrations (mg/kg) in anthropogenic sediments with increasing distance from the formerly old mining area. Results are shown for the eight study sites. The box indicates the 1st and 3rd quartiles, and the dark line indicates the median value for each site. Outliers are shown as black dots


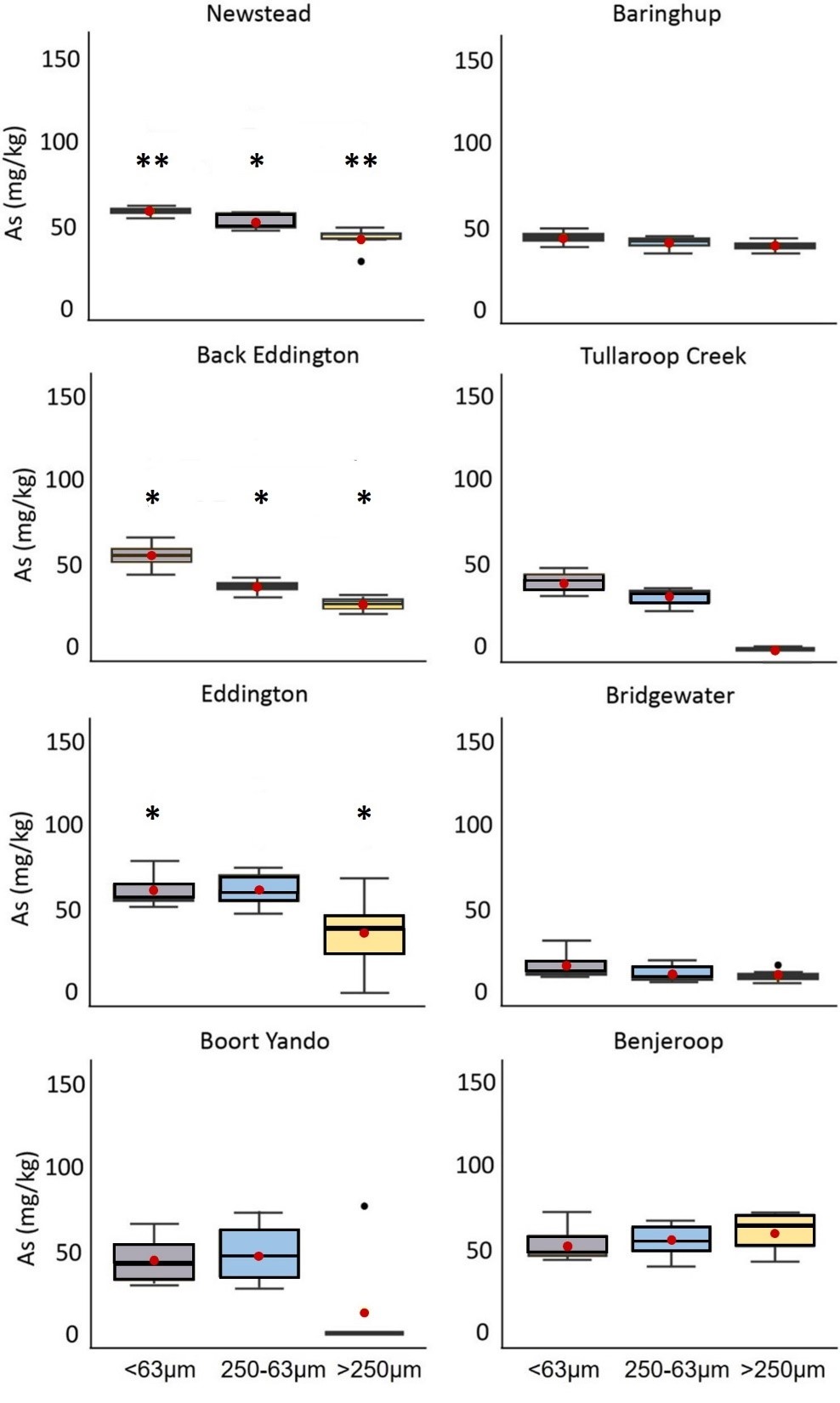


**Fig. S9**: Box and whisker plot of arsenic concentrations by p-XRF (mg/kg and g/kg) in original floodplain sediments from p-XRF measurements for the >250μm, 250-63μm and <63μm size fractions obtained for the eight river-bank deposits. The box indicates the 1st and 3rd quartiles, and the dark line indicates the median. The mean is indicated with red dots. Significant figures are indicated with *(< 0.05) and **(< 0.01)


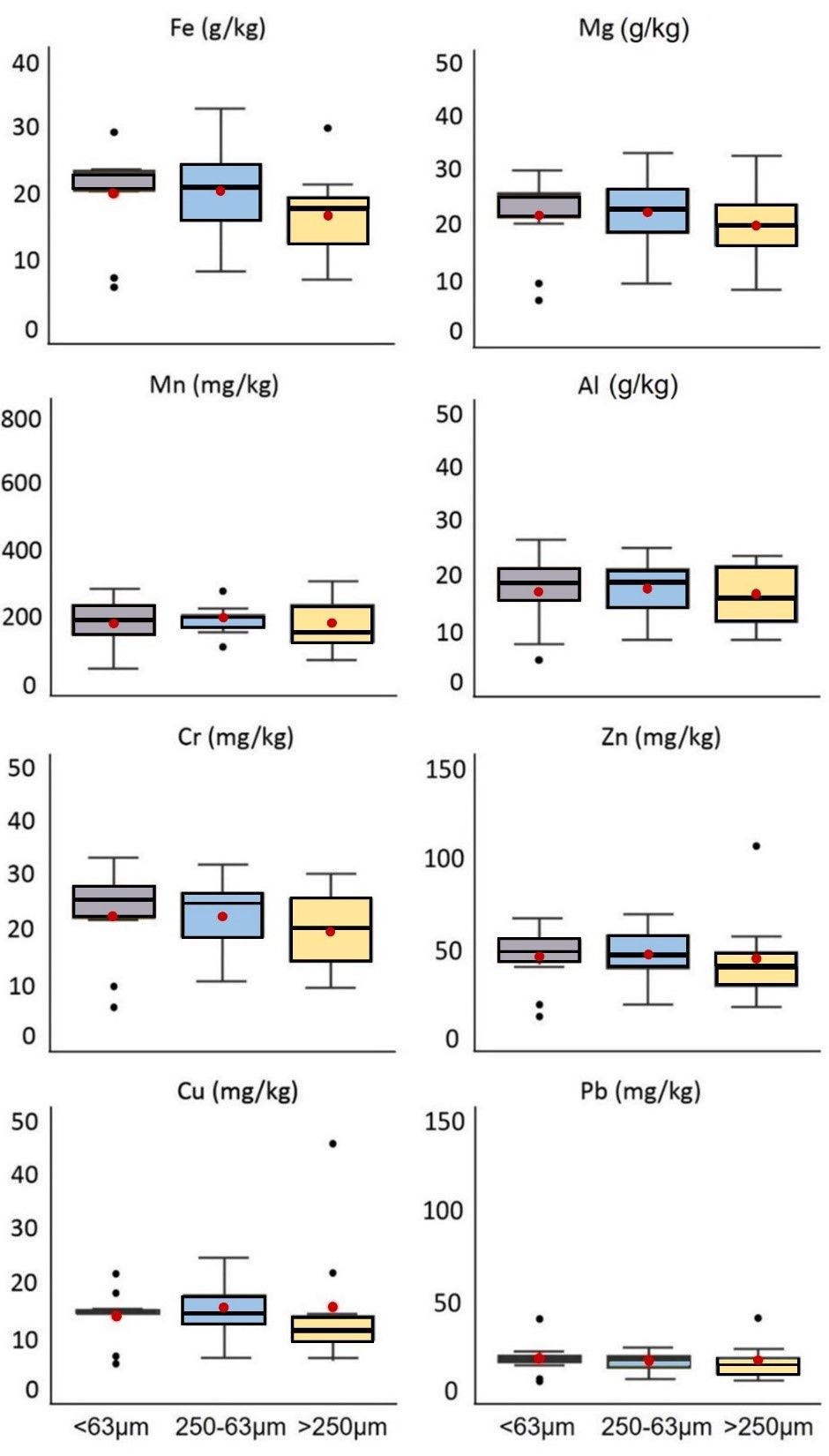


**Fig. S10**: Box and whisker plot of metal concentrations by ICP-MS (mg/kg and g/kg) for the >250μm, 250-63μm and <63μm size fractions. Anthropogenic sediments from Back Eddington. The box indicates the 1st and 3rd quartiles, and the dark line indicates the median. The mean is indicated by red dot. The mean is indicated with red dots. Significant figures are indicated with *(< 0.05) and **(< 0.01)


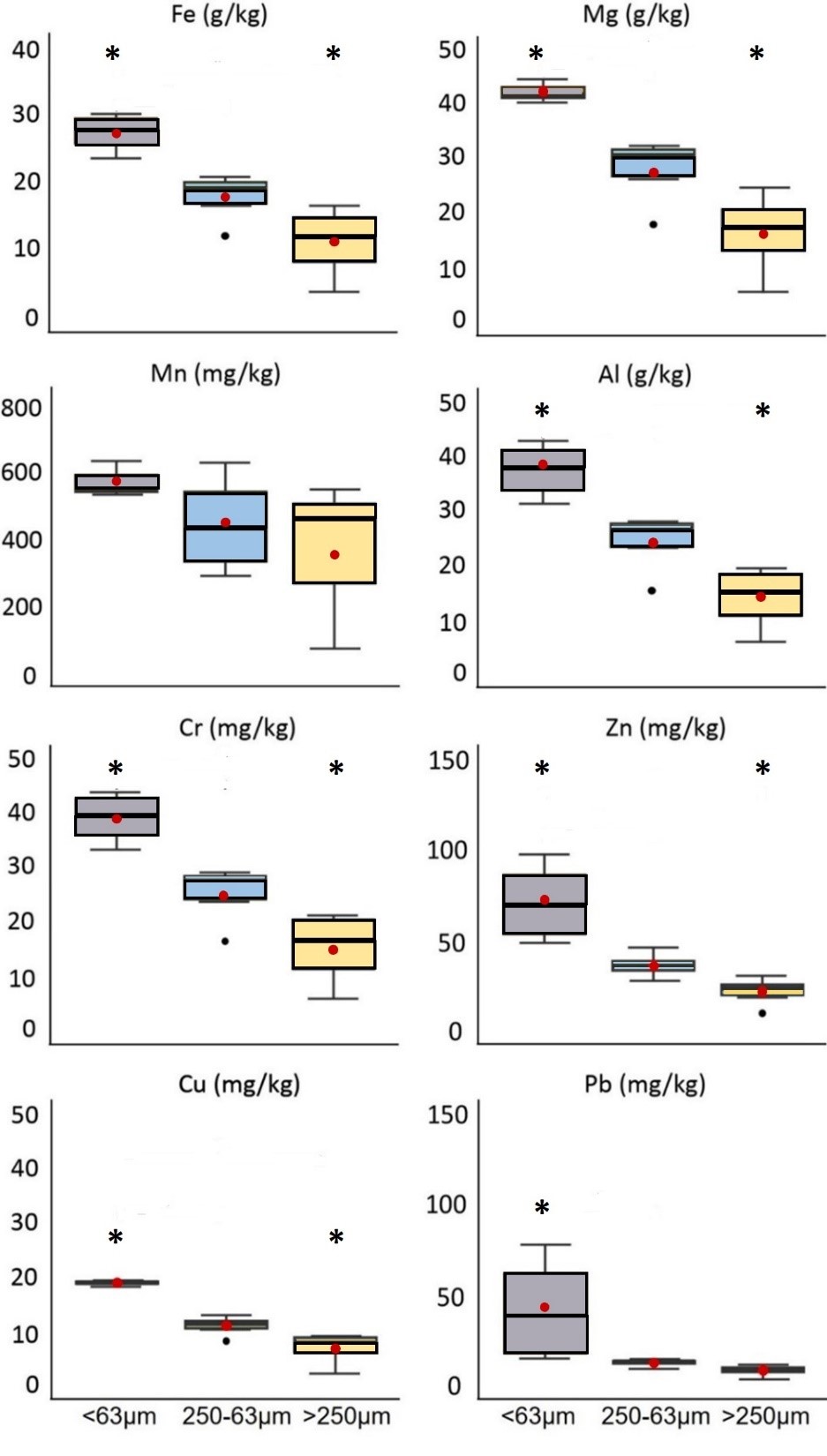


**Fig. S11**: Box and whisker plot of metal concentrations by ICP-MS (mg/kg and g/kg) for the >250μm, 250-63μm and <63μm size fractions. Original floodplain sediments from Back Eddington. The box indicates the 1st and 3rd quartiles, and the dark line indicates the median. The mean is indicated by red dots. The mean is indicated with red dots. Significant figures are indicated with *(< 0.05) and **(< 0.01)


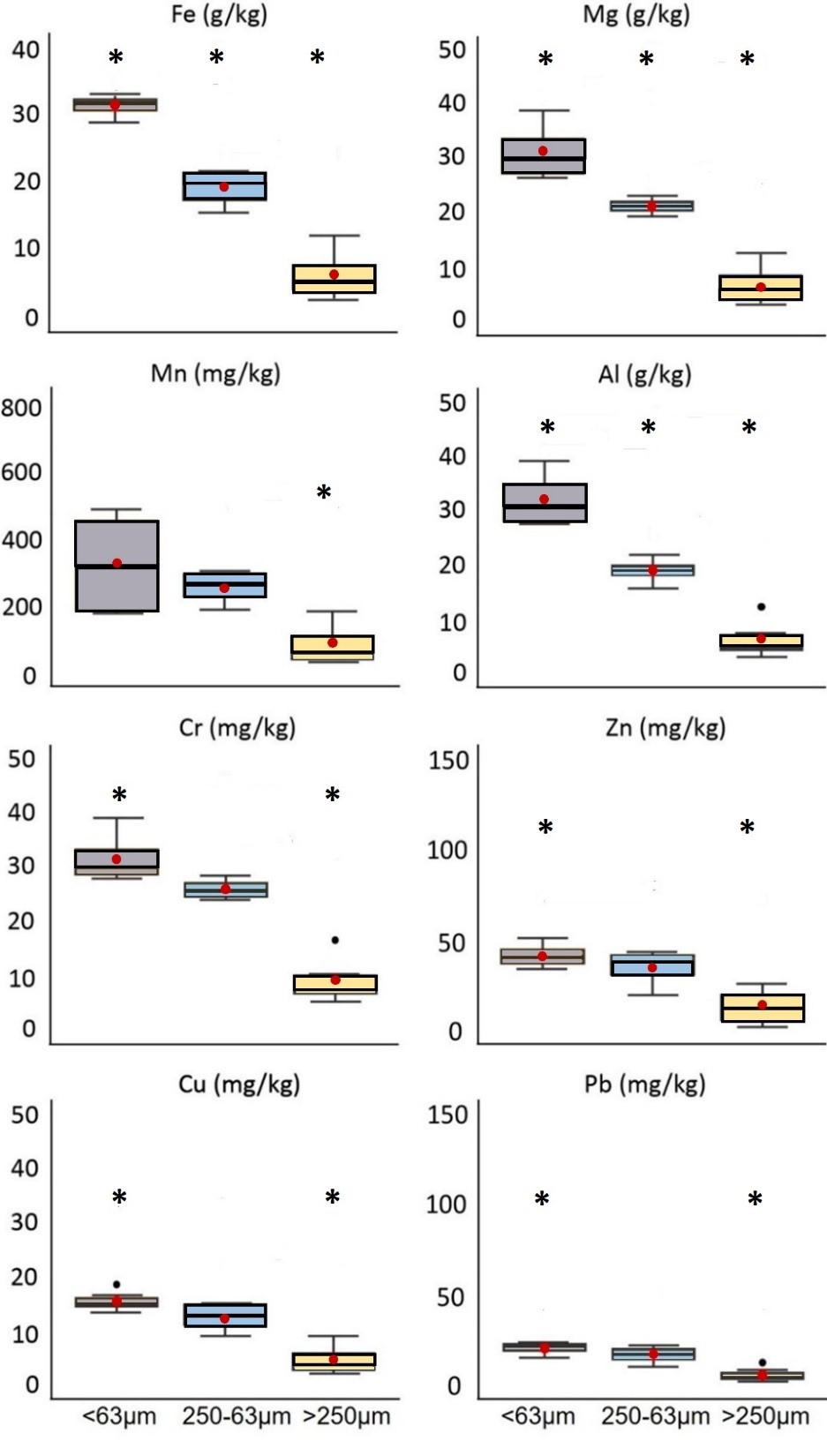


**Fig. S12**: Box and whisker plot of metal concentrations by ICP-MS (mg/kg and g/kg) for the >250μm, 250-63μm and <63μm size fractions. Anthropogenic sediments from Bridgewater. The box indicates the 1st and 3rd quartiles, and the dark line indicates the median. The mean is indicated by red dots The mean is indicated with red dots. Significant figures are indicated with *(< 0.05) and **(< 0.01)


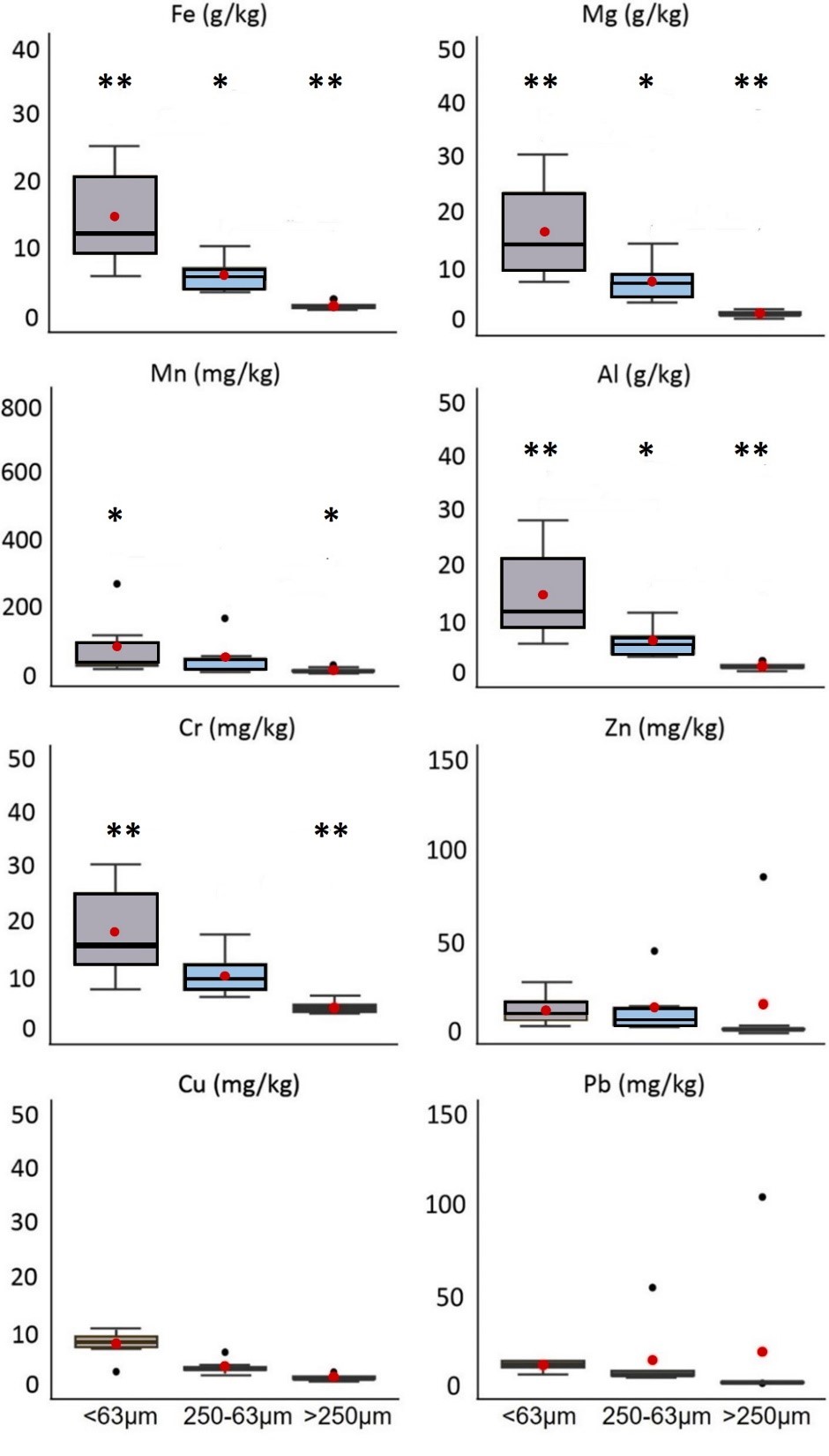


**Fig. S13**: Box and whisker plot of metal concentrations by ICP-MS (mg/kg and g/kg) for the >250μm, 250-63μm and <63μm size fractions. Original floodplain sediments from Bridgewater. The box indicates the 1st and 3rd quartiles, and the dark line indicates the median. The mean is indicated by red dots. The mean is indicated with red dots. Significant figures are indicated with *(< 0.05) and **(< 0.01)


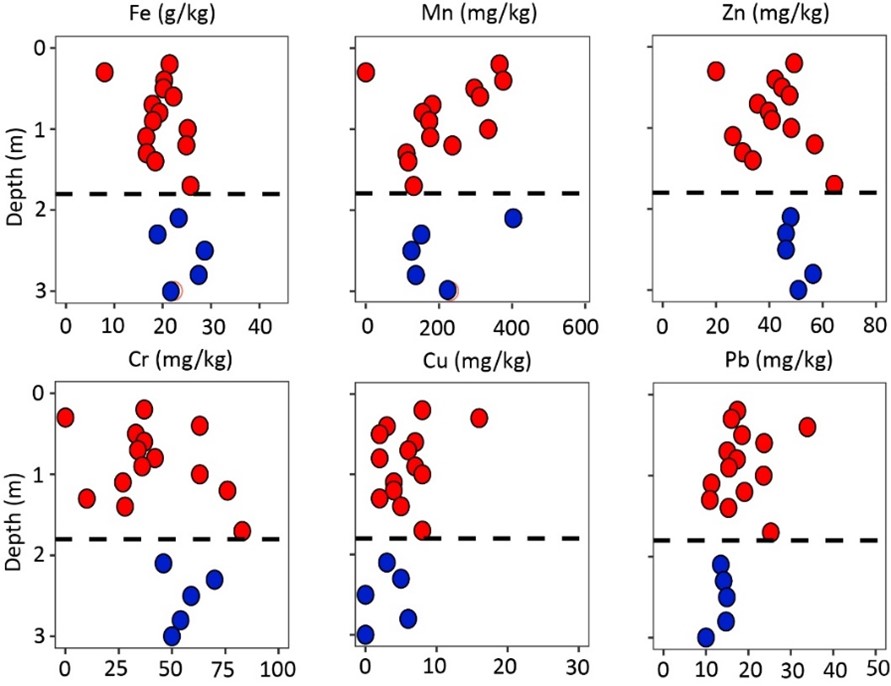


**Fig. S14**: Metals (Fe in g/kg) profiles for Newstead river-bank deposits along the Loddon River from p-XRF analysis. Profiles extend through the anthropic sediments through to original (relic) floodplain (boundary marked by horizontal dotted line)


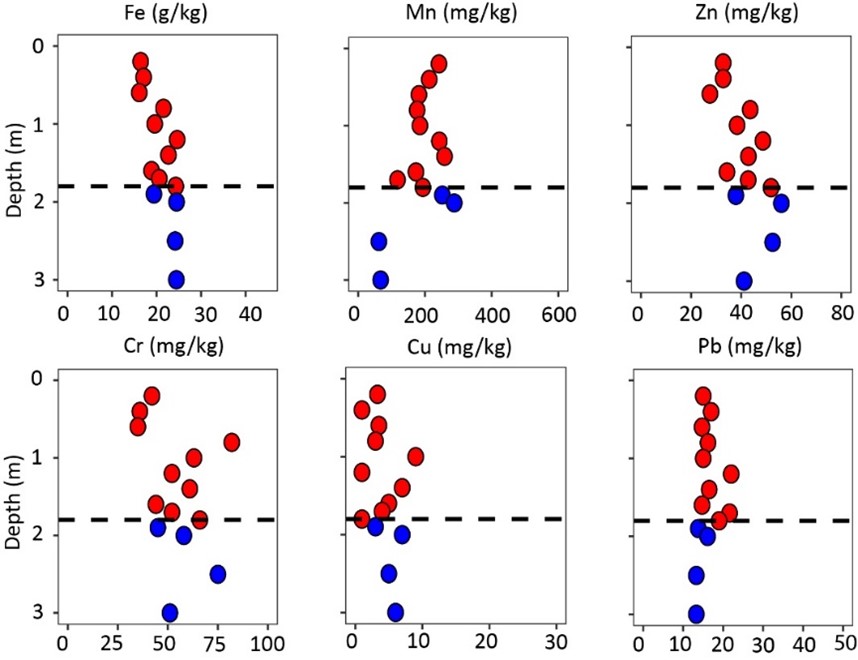


**Fig. S15**: Metals (Fe in g/kg) profiles for Baringhup river-bank deposits along the Loddon River from p-XRF analysis. Profiles extend through the anthropic sediments through to original (relic) floodplain (boundary marked by horizontal dotted line)


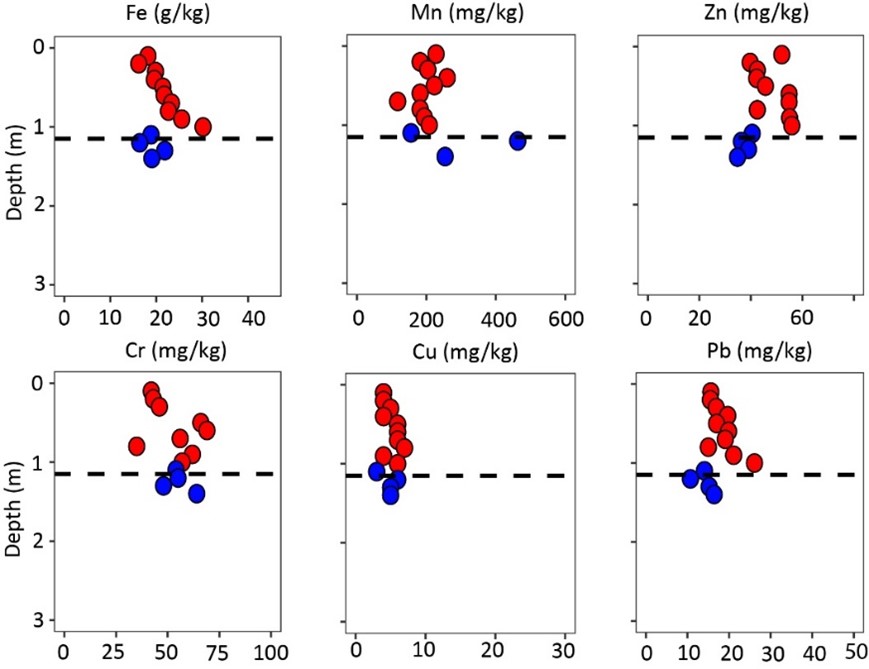


**Fig. S16**: Metals (Fe in g/kg) profiles for Back Eddington river-bank deposits along the Loddon River from p-XRF analysis. Profiles extend through the anthropic sediments through to original (relic) floodplain (boundary marked by horizontal dotted line)


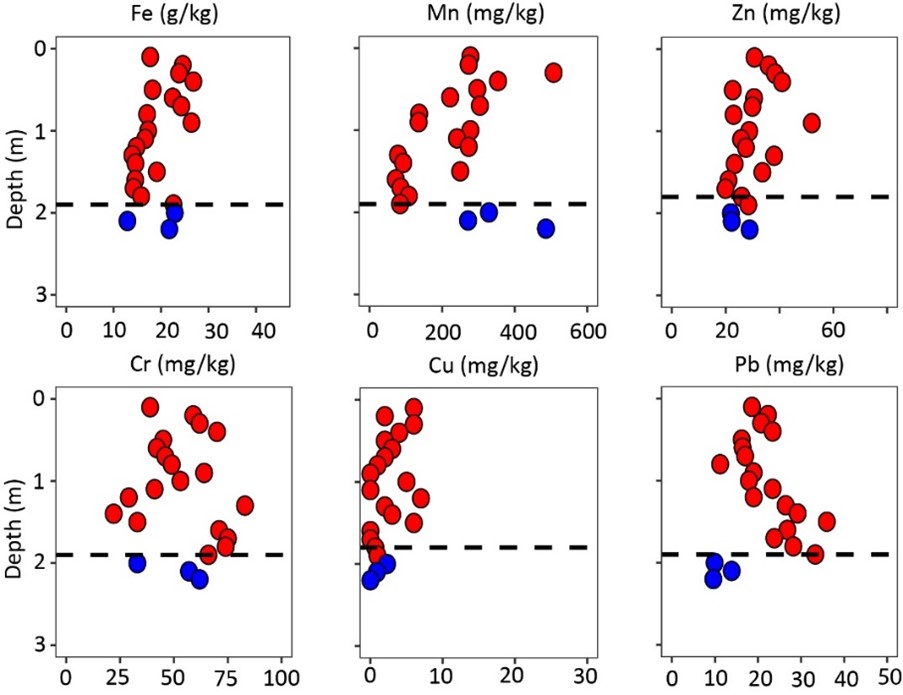


**Fig. S17**: Metals (Fe in g/kg) profiles for Tullaroop Creek river-bank deposits along the Loddon River from p-XRF analysis. Profiles extend through the anthropic sediments through to original (relic) floodplain (boundary marked by horizontal dotted line)


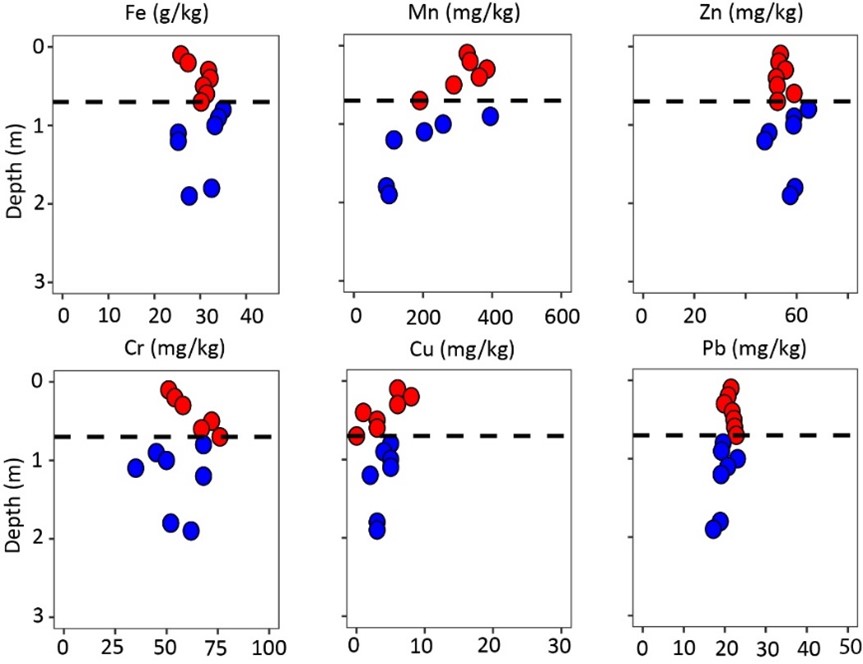


**Fig. S18**: Metals (Fe in g/kg) profiles for Eddington river-bank deposits along the Loddon River from p-XRF analysis. Profiles extend through the anthropic sediments through to original (relic) floodplain (boundary marked by horizontal dotted line)


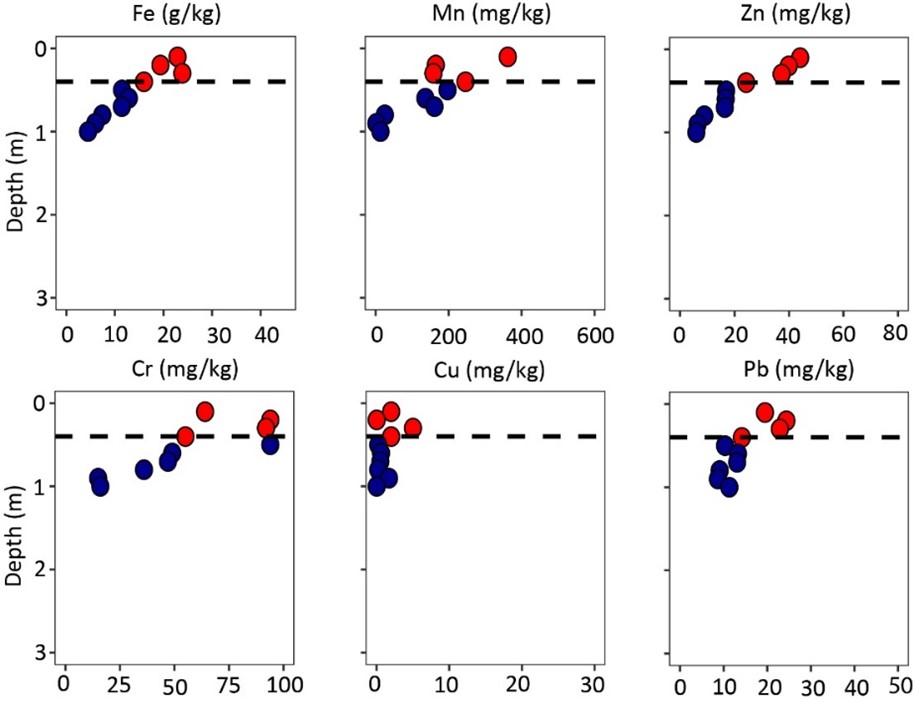


**Fig. S19**: Metals (Fe in g/kg) profiles for Bridgewater river-bank deposits along the Loddon River from p-XRF analysis. Profiles extend through the anthropic sediments through to original (relic) floodplain (boundary marked by horizontal dotted line)


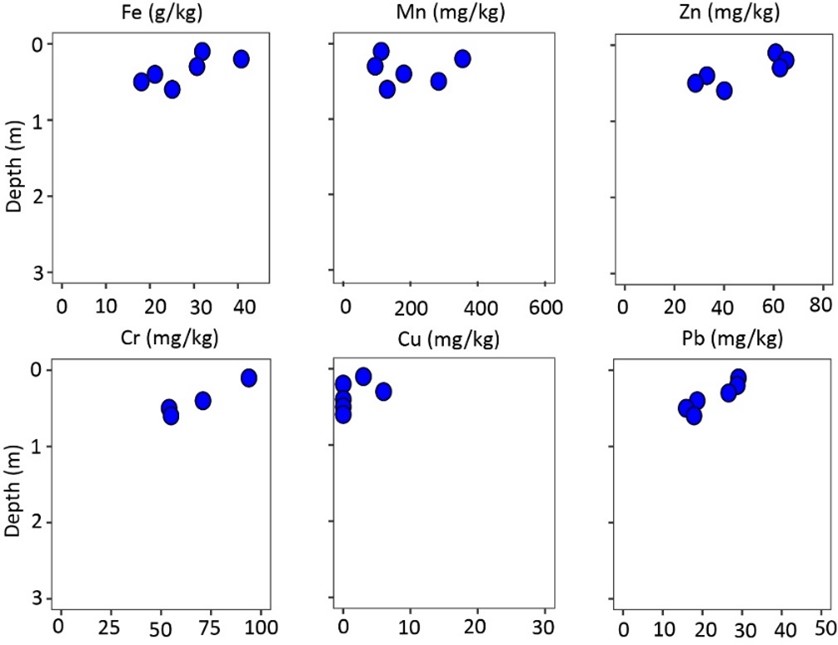


**Fig. S20**: Metals (Fe in g/kg) profiles for Boort Yando river-bank deposits along the Loddon River from p-XRF analysis. Profiles extend through the original (relic) floodplain


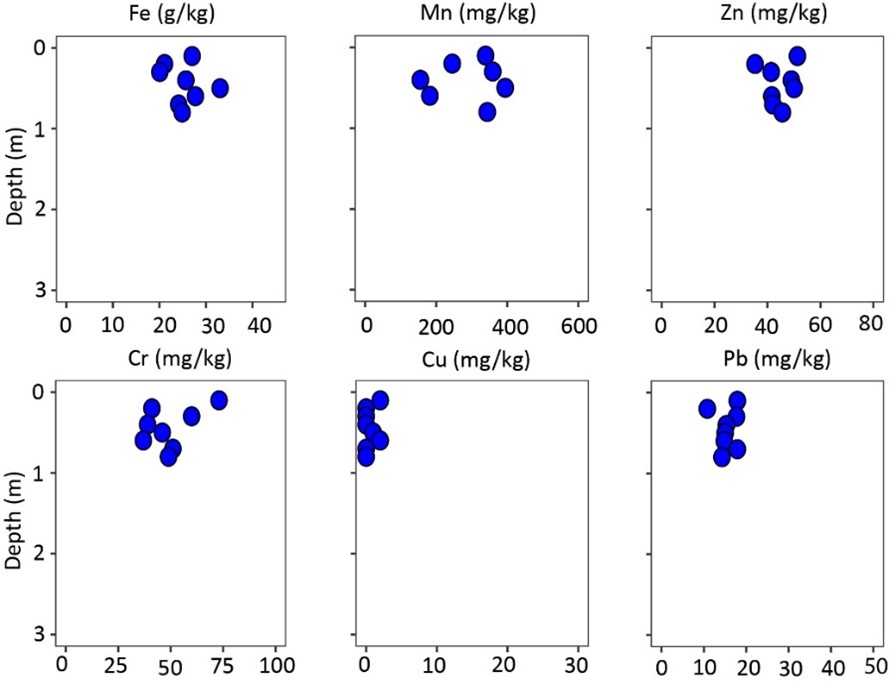


**Fig. S21**: Metals (Fe in g/kg) profiles for Benjeroop river-bank deposits along the Loddon River from p-XRF analysis. Profiles extend through the original (relic) floodplain


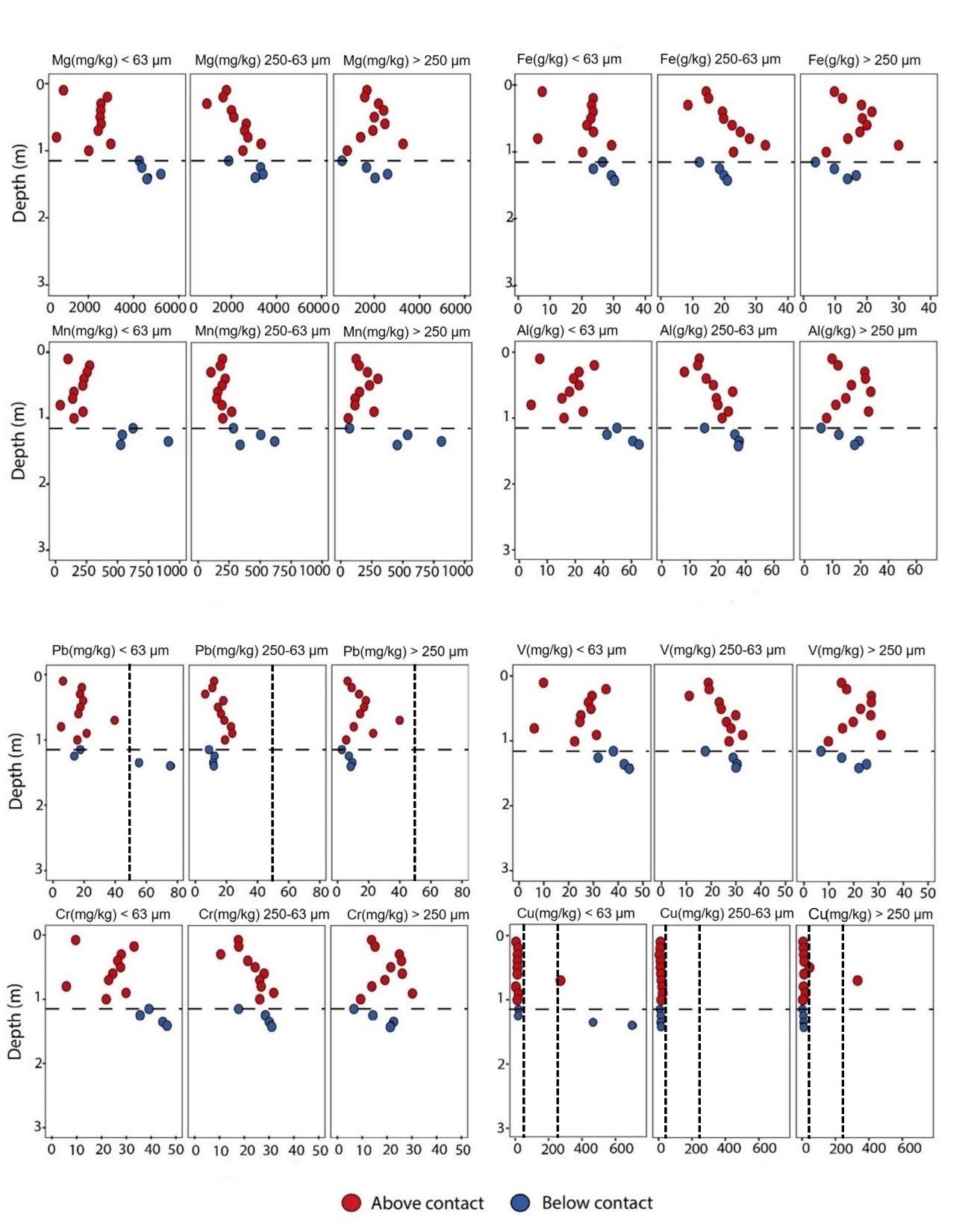


**Fig. S22**: Metals (Fe and Al in g/kg) profiles for Back Eddington river-bank deposits along the Loddon River from ICP-MS analysis. Profiles extend through the anthropic sediments through to original (relic) floodplain (boundary marked by horizontal dotted line). Vertical dotted lines represent the low level (50ppm) for Pb and the low level (65ppm) and high level (270ppm) for Cu of the interim sediment quality guidelines (ISQG) for Australia and New Zealand


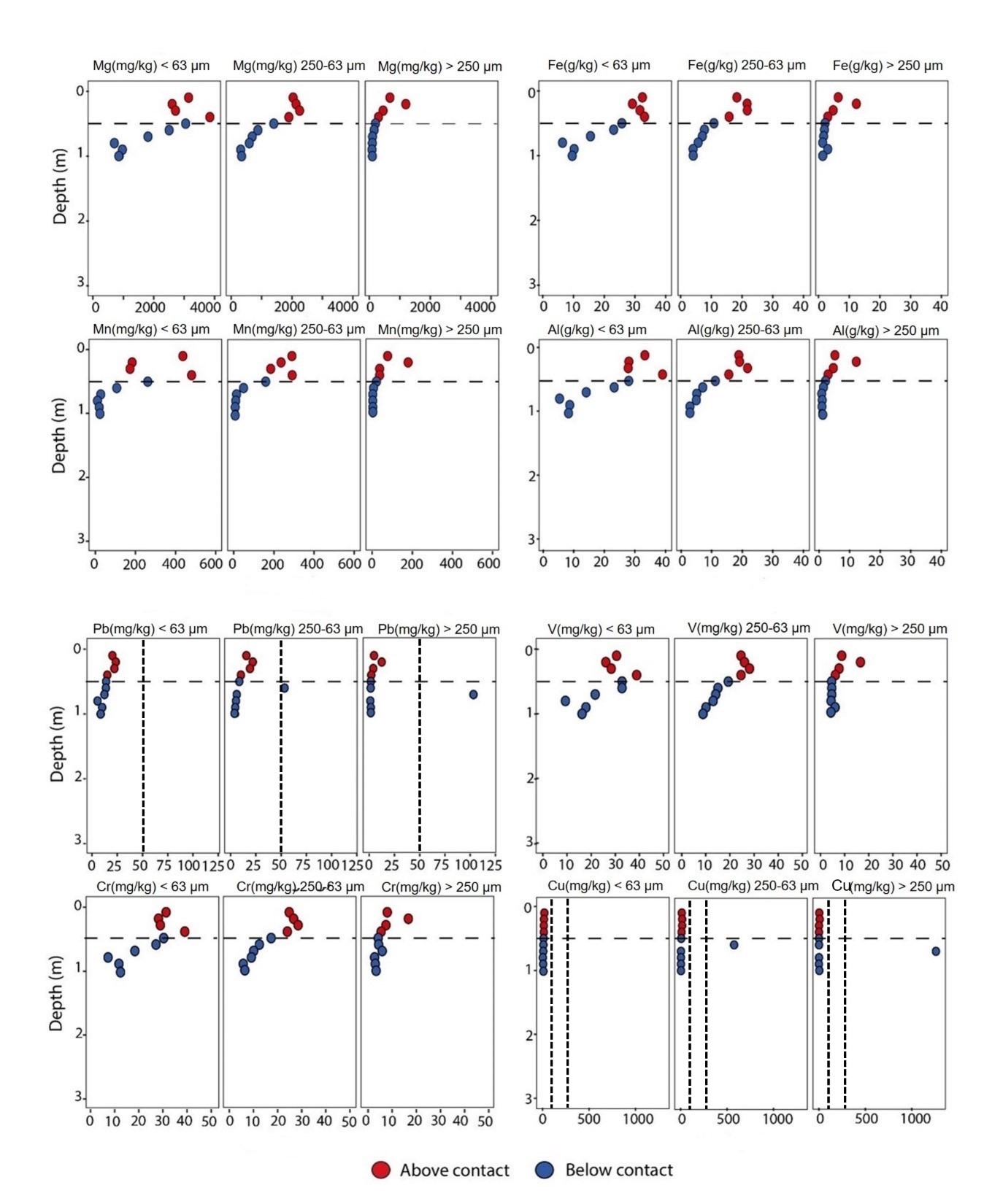


**Fig. S23**: Metals (Fe and Al in g/kg) profiles for Bridgewater river-bank deposits along the Loddon River from ICP-MS analysis. Profiles extend through the anthropic sediments through to original (relic) floodplain (boundary marked by horizontal dotted line). Vertical dotted lines represent the low level (50ppm) for Pb and the low level (65ppm) and high level (270ppm) for Cu of the interim sediment quality guidelines (ISQG) for Australia and New Zealand


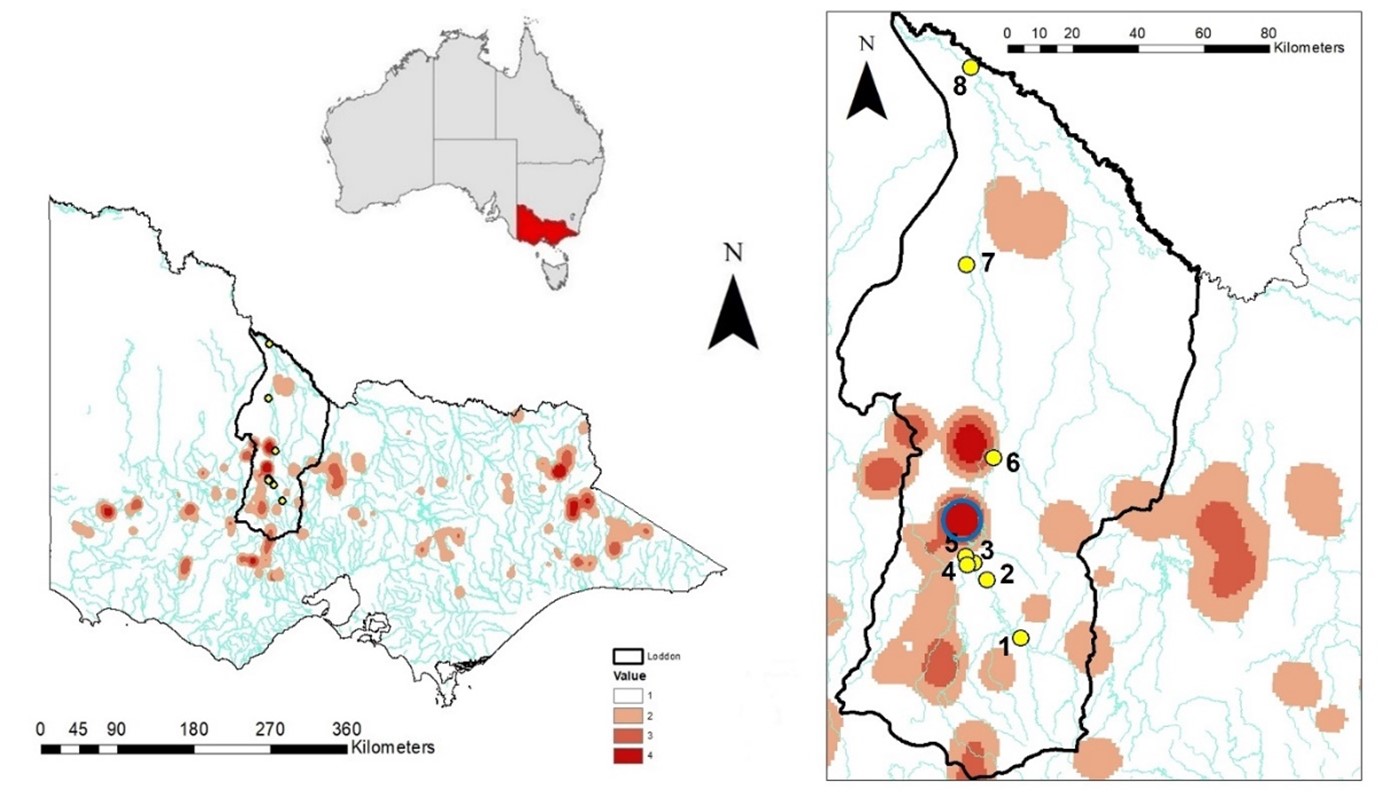


**Fig. S24**: The Kernel Density map [stretched to a low (white)-high (red) value] for arsenic clustering around Victoria, Australia. using ArcMap 10.6.1. The dataset was obtained from GeoVic database. Yellow circles = investigated sites. For name of sites refer to Figure 1

**Table S1**: Concentrations (mg/kg) of metals (p-XRF) in river-bank sediment deposits from eight sites along the Loddon River. Samples analysed every 10 cm

|  | As (mg/kg) | Mn (mg/kg) | Fe (mg/kg) | Cu (mg/kg) | Cr (mg/kg) | Zn (mg/kg) | Pb  (mg/kg) | Deposit | Depth (m) |
| --- | --- | --- | --- | --- | --- | --- | --- | --- | --- |
| Newstead | 22 | 366 | 21410 | 8 | 37 | 49 | 17 | Anthropogenic sediments | 0.2 |
| Newstead | 19 | 0 | 8008 | 16 | 0 | 20 | 16 | Anthropogenic sediments | 0.3 |
| Newstead | 24 | 376 | 20297 | 3 | 63 | 42 | 34 | Anthropogenic sediments | 0.4 |
| Newstead | 24 | 297 | 20149 | 2 | 33 | 45 | 19 | Anthropogenic sediments | 0.5 |
| Newstead | 27 | 312 | 22199 | 7 | 37 | 48 | 24 | Anthropogenic sediments | 0.6 |
| Newstead | 39 | 182 | 17908 | 6 | 34 | 36 | 15 | Anthropogenic sediments | 0.7 |
| Newstead | 26 | 155 | 19326 | 2 | 42 | 40 | 17 | Anthropogenic sediments | 0.8 |
| Newstead | 20 | 173 | 17999 | 7 | 36 | 41 | 15 | Anthropogenic sediments | 0.9 |
| Newstead | 48 | 335 | 25163 | 8 | 63 | 48 | 24 | Anthropogenic sediments | 1 |
| Newstead | 26 | 176 | 16599 | 4 | 27 | 26 | 11 | Anthropogenic sediments | 1.1 |
| Newstead | 41 | 237 | 24876 | 4 | 76 | 57 | 19 | Anthropogenic sediments | 1.2 |
| Newstead | 25 | 111 | 16673 | 2 | 10 | 30 | 11 | Anthropogenic sediments | 1.3 |
| Newstead | 28 | 116 | 18472 | 5 | 28 | 34 | 15 | Anthropogenic sediments | 1.4 |
| Newstead | 33 | 131 | 25726 | 8 | 83 | 64 | 25 | Anthropogenic sediments | 1.7 |
| Newstead | 24 | 403 | 23265 | 3 | 46 | 48 | 14 | Original floodplain | 2.1 |
| Newstead | 4 | 152 | 18908 | 5 | 70 | 46 | 14 | Original floodplain | 2.3 |
| Newstead | 8 | 125 | 28678 | 0 | 59 | 46 | 15 | Original floodplain | 2.5 |
| Newstead | 6 | 137 | 27424 | 6 | 54 | 56 | 15 | Original floodplain | 2.8 |
| Newstead | 6 | 230 | 22301 | 0 | 50 | 51 | 10 | Original floodplain | 3 |
| Baringhup | 17 | 242 | 16410 | 3 | 42 | 33 | 15 | Anthropogenic sediments | 0 |
| Baringhup | 16 | 213 | 17108 | 1 | 36 | 33 | 17 | Anthropogenic sediments | 0.2 |
| Baringhup | 23 | 182 | 16112 | 4 | 35 | 28 | 15 | Anthropogenic sediments | 0.4 |
| Baringhup | 29 | 177 | 21534 | 3 | 82 | 44 | 16 | Anthropogenic sediments | 0.6 |
| Baringhup | 39 | 185 | 19606 | 9 | 63 | 38 | 15 | Anthropogenic sediments | 0.8 |
| Baringhup | 65 | 243 | 24631 | 1 | 52 | 49 | 22 | Anthropogenic sediments | 1 |
| Baringhup | 72 | 259 | 22641 | 7 | 61 | 43 | 17 | Anthropogenic sediments | 1.2 |
| Baringhup | 50 | 173 | 18875 | 5 | 44 | 34 | 15 | Anthropogenic sediments | 1.4 |
| Baringhup | 41 | 118 | 20615 | 4 | 52 | 43 | 22 | Anthropogenic sediments | 1.6 |
| Baringhup | 29 | 194 | 24314 | 1 | 66 | 52 | 19 | Original floodplain | 1.8 |
| Baringhup | 8 | 252 | 19407 | 3 | 45 | 38 | 14 | Original floodplain | 1.9 |
| Baringhup | 6 | 288 | 24477 | 7 | 58 | 56 | 16 | Original floodplain | 2 |
| Baringhup | 7 | 62 | 24182 | 5 | 75 | 53 | 13 | Original floodplain | 2.5 |
| Baringhup | 5 | 67 | 24432 | 6 | 51 | 41 | 13 | Original floodplain | 3 |
| B. Eddington | 49 | 228 | 18183 | 4 | 42 | 52 | 16 | Anthropogenic sediments | 0.1 |
| B. Eddington | 22 | 182 | 16193 | 4 | 43 | 40 | 16 | Anthropogenic sediments | 0.2 |
| B. Eddington | 27 | 204 | 19865 | 5 | 46 | 43 | 17 | Anthropogenic sediments | 0.3 |
| B. Eddington | 25 | 260 | 19579 | 4 | 106 | 42 | 20 | Anthropogenic sediments | 0.4 |
| B. Eddington | 31 | 223 | 21475 | 6 | 66 | 46 | 17 | Anthropogenic sediments | 0.5 |
| B. Eddington | 35 | 182 | 21729 | 6 | 69 | 55 | 20 | Anthropogenic sediments | 0.6 |
| B. Eddington | 83 | 117 | 23306 | 6 | 56 | 55 | 19 | Anthropogenic sediments | 0.7 |
| B. Eddington | 121 | 182 | 22657 | 7 | 35 | 43 | 15 | Anthropogenic sediments | 0.8 |
| B. Eddington | 60 | 194 | 25544 | 4 | 62 | 55 | 21 | Anthropogenic sediments | 0.9 |
| B. Eddington | 63 | 208 | 30182 | 6 | 57 | 56 | 26 | Anthropogenic sediments | 1 |
| B. Eddington | 19 | 156 | 18860 | 3 | 54 | 41 | 14 | Original floodplain | 1.15 |
| B. Eddington | 5 | 463 | 16402 | 6 | 55 | 36 | 11 | Original floodplain | 1.25 |
| B. Eddington | 7 | 780 | 21847 | 5 | 48 | 39 | 15 | Original floodplain | 1.35 |
| B. Eddington | 4 | 254 | 19035 | 5 | 64 | 35 | 16 | Original floodplain | 1.4 |
| Benjeroop | 8 | 339 | 27012 | 2 | 73 | 51 | 18 | Original floodplain | 0 |
| Benjeroop | 6 | 245 | 21117 | 0 | 41 | 35 | 11 | Original floodplain | 0.1 |
| Benjeroop | 4 | 359 | 20082 | 0 | 60 | 41 | 18 | Original floodplain | 0.2 |
| Benjeroop | 6 | 155 | 25704 | 0 | 39 | 49 | 15 | Original floodplain | 0.3 |
| Benjeroop | 4 | 394 | 33040 | 1 | 46 | 50 | 15 | Original floodplain | 0.4 |
| Benjeroop | 3 | 182 | 27752 | 2 | 37 | 42 | 15 | Original floodplain | 0.5 |
| Benjeroop | 6 | 639 | 24109 | 0 | 51 | 42 | 18 | Original floodplain | 0.6 |
| Benjeroop | 4 | 344 | 24908 | 0 | 49 | 46 | 14 | Original floodplain | 0.7 |
| Eddington | 37 | 328 | 25794 | 6 | 51 | 54 | 22 | Anthropogenic sediments | 0.1 |
| Eddington | 35 | 336 | 27367 | 8 | 54 | 53 | 21 | Anthropogenic sediments | 0.2 |
| Eddington | 35 | 385 | 31798 | 6 | 58 | 56 | 20 | Anthropogenic sediments | 0.3 |
| Eddington | 34 | 363 | 32161 | 1 | 109 | 52 | 22 | Anthropogenic sediments | 0.4 |
| Eddington | 34 | 289 | 30671 | 3 | 72 | 52 | 22 | Anthropogenic sediments | 0.5 |
| Eddington | 35 | 668 | 31374 | 3 | 67 | 59 | 22 | Anthropogenic sediments | 0.6 |
| Eddington | 35 | 191 | 30180 | 0 | 76 | 53 | 23 | Original floodplain | 0.7 |
| Eddington | 40 | 702 | 34962 | 5 | 68 | 65 | 20 | Original floodplain | 0.8 |
| Eddington | 37 | 395 | 34005 | 4 | 45 | 59 | 19 | Original floodplain | 0.9 |
| Eddington | 32 | 258 | 33199 | 5 | 50 | 59 | 23 | Original floodplain | 1.0 |
| Eddington | 21 | 204 | 25214 | 5 | 35 | 49 | 21 | Original floodplain | 1.1 |
| Eddington | 25 | 116 | 25204 | 2 | 68 | 48 | 19 | Original floodplain | 1.2 |
| Eddington | 41 | 94 | 32464 | 3 | 52 | 59 | 19 | Original floodplain | 1.8 |
| Eddington | 22 | 102 | 27598 | 3 | 62 | 58 | 17 | Original floodplain | 1.9 |
| Bridgewater | 26 | 362 | 22887 | 2 | 64 | 44 | 20 | Anthropogenic sediments | 0.1 |
| Bridgewater | 76 | 165 | 19352 | 0 | 94 | 40 | 24 | Anthropogenic sediments | 0.2 |
| Bridgewater | 43 | 158 | 23851 | 5 | 92 | 37 | 23 | Anthropogenic sediments | 0.3 |
| Bridgewater | 4 | 246 | 15976 | 2 | 55 | 24 | 14 | Anthropogenic sediments | 0.4 |
| Bridgewater | 5 | 197 | 11456 | 0.2 | 94 | 17 | 10 | Original floodplain | 0.5 |
| Bridgewater | 4 | 136 | 12848 | 0.6 | 49 | 17 | 13 | Original floodplain | 0.6 |
| Bridgewater | 4 | 161 | 11427 | 0.5 | 47 | 16 | 13 | Original floodplain | 0.7 |
| Bridgewater | 4 | 25 | 7419 | 0.2 | 36 | 9 | 9 | Original floodplain | 0.8 |
| Bridgewater | 3 | 2 | 5941 | 1.7 | 15 | 6 | 9 | Original floodplain | 0.9 |
| Bridgewater | 1 | 13 | 4462 | 0 | 16 | 6 | 11 | Original floodplain | 1.0 |
| Boort-Yando | 13 | 113 | 31872 | 3 | 94 | 61 | 29 | Original floodplain | 0.1 |
| Boort-Yando | 53 | 355 | 40781 | 0 | 106 | 65 | 29 | Original floodplain | 0.2 |
| Boort-Yando | 19 | 95 | 30700 | 6 | 110 | 63 | 27 | Original floodplain | 0.3 |
| Boort-Yando | 11 | 180 | 21158 | 0 | 71 | 33 | 19 | Original floodplain | 0.4 |
| Boort-Yando | 7 | 284 | 18089 | 0 | 54 | 29 | 16 | Original floodplain | 0.5 |
| Boort-Yando | 9 | 131 | 25053 | 0 | 55 | 40 | 18 | Original floodplain | 0.6 |
| Tullaroop Ck | 52 | 278 | 17719 | 6 | 39 | 31 | 19 | Anthropogenic sediments | 0.1 |
| Tullaroop Ck | 37 | 273 | 24595 | 2 | 59 | 36 | 22 | Anthropogenic sediments | 0.2 |
| Tullaroop Ck | 40 | 507 | 23752 | 6 | 62 | 38 | 21 | Anthropogenic sediments | 0.3 |
| Tullaroop Ck | 38 | 354 | 26786 | 4 | 70 | 41 | 23 | Anthropogenic sediments | 0.4 |
| Tullaroop Ck | 25 | 297 | 18198 | 2 | 45 | 23 | 16 | Anthropogenic sediments | 0.5 |
| Tullaroop Ck | 30 | 222 | 22416 | 3 | 42 | 31 | 16 | Anthropogenic sediments | 0.6 |
| Tullaroop Ck | 47 | 304 | 24285 | 2 | 46 | 30 | 17 | Anthropogenic sediments | 0.7 |
| Tullaroop Ck | 35 | 136 | 17044 | 1 | 49 | 23 | 11 | Anthropogenic sediments | 0.8 |
| Tullaroop Ck | 21 | 135 | 26427 | 0 | 64 | 52 | 19 | Anthropogenic sediments | 0.9 |
| Tullaroop Ck | 66 | 278 | 17271 | 5 | 53 | 29 | 18 | Anthropogenic sediments | 1.0 |
| Tullaroop Ck | 129 | 241 | 16623 | 0 | 41 | 26 | 23 | Anthropogenic sediments | 1.1 |
| Tullaroop Ck | 146 | 273 | 14810 | 7 | 29 | 28 | 19 | Anthropogenic sediments | 1.2 |
| Tullaroop Ck | 135 | 78 | 13919 | 2 | 83 | 38 | 26 | Anthropogenic sediments | 1.3 |
| Tullaroop Ck | 223 | 92 | 14586 | 3 | 22 | 23 | 29 | Anthropogenic sediments | 1.4 |
| Tullaroop Ck | 312 | 250 | 19095 | 6 | 33 | 34 | 36 | Anthropogenic sediments | 1.5 |
| Tullaroop Ck | 76 | 72 | 14546 | 0 | 71 | 21 | 27 | Anthropogenic sediments | 1.6 |
| Tullaroop Ck | 44 | 85 | 14174 | 0 | 75 | 20 | 24 | Anthropogenic sediments | 1.7 |
| Tullaroop Ck | 34 | 108 | 15795 | 1 | 74 | 26 | 28 | Anthropogenic sediments | 1.8 |
| Tullaroop Ck | 36 | 84 | 22637 | 1 | 66 | 28 | 33 | Anthropogenic sediments | 1.9 |
| Tullaroop Ck | 13 | 329 | 22901 | 2 | 33 | 22 | 10 | Original floodplain | 2.0 |
| Tullaroop Ck | 10 | 271 | 12914 | 1 | 57 | 22 | 14 | Original floodplain | 2.1 |
| Tullaroop Ck | 6 | 486 | 21733 | 0 | 62 | 29 | 10 | Original floodplain | 2.2 |

**Table S2**: Concentrations (mg/kg) of metals (ICP-MS) in Back Eddington and Bridgewater deposits from the Loddon River. Samples analysed every 10 cm

|  |  | Mg |  |  | Al |  |  | Ca |  |  | V |  |  |
| --- | --- | --- | --- | --- | --- | --- | --- | --- | --- | --- | --- | --- | --- |
| ID | Depth | > 250μm | 250-63μm | < 63μm | > 250μm | 250-63μm | < 63μm | > 250μm | 250-63μm | < 63μm | > 250μm | 250-63μm | < 63μm |
| 180 | 0.1 | 1677.1 | 1783.5 | 898.6 | 9867.5 | 13362.3 | 7277.1 | 1083.9 | 1774.1 | 720.1 | 15.1 | 18.9 | 9.9 |
| 181 | 0.2 | 1578.8 | 1639.3 | 2843.0 | 12001.7 | 12847.4 | 26793.9 | 1404.4 | 1275.6 | 2070.3 | 17.2 | 19.2 | 35.1 |
| 182 | 0.3 | 2189.3 | 920.4 | 2556.6 | 21505.7 | 8013.6 | 21318.9 | 1545.1 | 642.9 | 1814.2 | 27.1 | 11.2 | 29.4 |
| 183 | 0.4 | 2410.7 | 2011.2 | 2547.7 | 21781.0 | 15827.6 | 19356.1 | 1880.6 | 1525.2 | 1751.9 | 27.1 | 23.3 | 28.0 |
| 184 | 0.5 | 2004.7 | 2110.1 | 2517.8 | 16772.3 | 18371.8 | 21312.8 | 1449.6 | 1368.4 | 1497.6 | 22.8 | 24.1 | 29.0 |
| 185 | 0.6 | 2477.1 | 2662.6 | 2572.3 | 23676.3 | 25268.3 | 17919.5 | 1267.3 | 1357.4 | 1165.3 | 26.9 | 29.9 | 24.9 |
| 186 | 0.7 | 1944.6 | 2585.7 | 2432.5 | 14764.8 | 19413.8 | 15252.7 | 971.6 | 1333.9 | 1063.1 | 19.8 | 26.2 | 24.5 |
| 187 | 0.8 | 1392.3 | 2729.5 | 588.5 | 11198.6 | 20015.9 | 4247.3 | 922.8 | 1469.5 | 200.0 | 15.5 | 27.9 | 6.2 |
| 188 | 0.9 | 3276.7 | 3312.6 | 2990.5 | 22894.0 | 23740.4 | 22806.6 | 1445.1 | 1639.1 | 1212.2 | 31.1 | 32.7 | 31.3 |
| 189 | 1 | 804.1 | 2517.7 | 2019.2 | 7898.1 | 21522.8 | 15972.0 | 383.2 | 1217.7 | 832.4 | 9.8 | 27.3 | 22.4 |
| 190 | 1.15 | 574.3 | 1882.9 | 4250.7 | 5931.9 | 15263.2 | 34834.9 | 509.8 | 1642.5 | 4011.2 | 6.8 | 17.6 | 38.1 |
| 191 | 1.25 | 1655.5 | 3297.7 | 4367.4 | 12253.9 | 26063.7 | 31304.7 | 1634.3 | 3140.3 | 4583.7 | 15.2 | 28.9 | 31.9 |
| 192 | 1.35 | 2587.6 | 3397.1 | 5207.9 | 19492.4 | 27591.9 | 40520.9 | 2269.0 | 2871.6 | 4419.3 | 25.2 | 30.5 | 42.4 |
| 193 | 1.4 | 2037.1 | 3118.7 | 4665.5 | 17942.0 | 27737.4 | 42975.8 | 1643.8 | 2372.6 | 3501.3 | 22.1 | 30.4 | 44.8 |
|  |  |  |  |  |  |  |  |  |  |  |  |  |  |
| 234 | 0.1 | 673.2 | 2036.7 | 3143.2 | 5228.0 | 18901.8 | 33222.4 | 1057.4 | 1999.9 | 2488.4 | 8.8 | 24.6 | 30.5 |
| 235 | 0.2 | 1197.1 | 2132.8 | 2604.1 | 12192.4 | 19108.1 | 28015.0 | 667.7 | 1265.8 | 1405.2 | 16.6 | 26.1 | 26.1 |
| 236 | 0.3 | 451.4 | 2250.7 | 2715.3 | 4706.5 | 21749.1 | 27810.0 | 138.7 | 1260.7 | 1289.6 | 7.7 | 28.2 | 28.3 |
| 237 | 0.4 | 297.1 | 1899.1 | 3844.5 | 2972.4 | 15579.5 | 39014.6 | 87.3 | 1230.9 | 2453.7 | 6.2 | 24.6 | 38.9 |
| 238 | 0.5 | 195.6 | 1398.9 | 3050.0 | 2125.3 | 11212.1 | 27960.5 | 11.4 | 835.6 | 2037.8 | 4.7 | 19.3 | 32.8 |
| 239 | 0.6 | 152.8 | 883.1 | 2505.3 | 1438.9 | 7105.3 | 23253.5 | -20.4 | 343.6 | 1222.4 | 4.7 | 15.0 | 32.8 |
| 240 | 0.7 | 102.7 | 694.4 | 1810.9 | 911.1 | 5114.9 | 14085.1 | -17.5 | 241.8 | 579.7 | 4.7 | 14.1 | 21.7 |
| 241 | 0.8 | 96.6 | 596.6 | 705.8 | 1075.3 | 5010.3 | 5360.0 | -40.2 | 318.5 | 186.6 | 4.4 | 13.0 | 9.3 |
| 242 | 0.9 | 85.5 | 314.4 | 973.3 | 993.0 | 2897.0 | 8699.0 | -77.8 | 17.4 | 122.0 | 6.0 | 10.1 | 17.8 |
| 243 | 1 | 94.6 | 362.1 | 902.2 | 916.4 | 2883.5 | 8168.1 | 42.3 | 12.8 | 168.5 | 4.3 | 9.4 | 16.3 |

|  |  | Cr |  |  | Mn |  |  | Fe |  |  | Co |  |  |
| --- | --- | --- | --- | --- | --- | --- | --- | --- | --- | --- | --- | --- | --- |
| ID | Depth | > 250μm | 250-63μm | < 63μm | > 250μm | 250-63μm | < 63μm | > 250μm | 250-63μm | < 63μm | > 250μm | 250-63μm | < 63μm |
| 180 | 0.1 | 13.8 | 17.6 | 9.6 | 124.3 | 197.6 | 101.7 | 9768.9 | 14275.6 | 7524.5 | 3.5 | 5.8 | 3.0 |
| 181 | 0.2 | 15.2 | 17.8 | 33.2 | 148.9 | 179.0 | 275.8 | 12290.2 | 15057.9 | 23559.5 | 4.5 | 5.9 | 9.2 |
| 182 | 0.3 | 25.0 | 10.5 | 27.9 | 216.2 | 103.3 | 255.6 | 18274.9 | 8503.9 | 23065.3 | 7.1 | 3.4 | 8.7 |
| 183 | 0.4 | 25.8 | 21.4 | 26.5 | 299.7 | 218.5 | 229.7 | 21542.1 | 19368.5 | 23520.8 | 10.4 | 7.9 | 8.8 |
| 184 | 0.5 | 21.5 | 24.4 | 27.7 | 232.2 | 194.0 | 220.4 | 18494.0 | 19749.1 | 22846.5 | 8.0 | 7.3 | 8.3 |
| 185 | 0.6 | 26.1 | 28.0 | 24.5 | 152.1 | 158.6 | 148.6 | 19923.8 | 22445.0 | 21598.1 | 7.8 | 7.9 | 7.8 |
| 186 | 0.7 | 19.2 | 26.2 | 22.9 | 116.6 | 151.3 | 137.9 | 17778.0 | 25003.2 | 23660.6 | 6.6 | 8.6 | 8.1 |
| 187 | 0.8 | 13.9 | 26.8 | 5.8 | 114.4 | 192.3 | 38.5 | 13966.2 | 27882.0 | 6125.1 | 5.3 | 9.9 | 2.1 |
| 188 | 0.9 | 30.2 | 31.9 | 29.9 | 268.6 | 270.5 | 222.0 | 29957.4 | 32880.4 | 29359.3 | 13.2 | 12.7 | 10.9 |
| 189 | 1 | 9.4 | 26.3 | 21.9 | 60.4 | 198.8 | 148.8 | 7162.2 | 22822.0 | 20254.1 | 2.9 | 9.4 | 7.4 |
| 190 | 1.15 | 6.7 | 17.7 | 39.1 | 70.7 | 284.8 | 625.0 | 3776.4 | 12123.5 | 26535.4 | 1.6 | 5.5 | 11.9 |
| 191 | 1.25 | 14.3 | 28.5 | 35.6 | 539.7 | 505.1 | 539.6 | 9718.1 | 18490.5 | 23610.3 | 6.6 | 8.4 | 9.4 |
| 192 | 1.35 | 22.6 | 30.1 | 44.7 | 812.5 | 617.3 | 910.1 | 16577.2 | 19816.5 | 29274.8 | 10.7 | 9.8 | 14.6 |
| 193 | 1.4 | 21.6 | 30.9 | 46.5 | 455.4 | 341.4 | 525.2 | 14285.2 | 20855.9 | 30350.6 | 8.6 | 8.7 | 13.1 |
|  |  |  |  |  |  |  |  |  |  |  |  |  |  |
| 234 | 0.1 | 7.9 | 24.9 | 31.3 | 76.5 | 289.8 | 436.3 | 6379.5 | 18316.0 | 32410.4 | 2.3 | 7.9 | 9.8 |
| 235 | 0.2 | 16.7 | 26.7 | 28.1 | 179.0 | 236.0 | 183.5 | 12365.5 | 21670.8 | 29166.6 | 8.8 | 10.5 | 7.9 |
| 236 | 0.3 | 7.4 | 28.5 | 28.9 | 37.4 | 184.0 | 172.8 | 4768.7 | 21689.8 | 31596.7 | 1.8 | 8.2 | 7.1 |
| 237 | 0.4 | 5.4 | 24.1 | 39.1 | 37.4 | 291.1 | 480.2 | 3010.1 | 15763.4 | 33120.3 | 1.3 | 8.4 | 11.5 |
| 238 | 0.5 | 4.2 | 17.4 | 30.3 | 21.4 | 159.2 | 261.5 | 2165.3 | 10854.2 | 25712.9 | 1.0 | 7.0 | 9.7 |
| 239 | 0.6 | 4.4 | 12.4 | 27.1 | 6.4 | 48.7 | 107.0 | 1960.7 | 7749.4 | 23051.8 | 0.6 | 3.2 | 6.6 |
| 240 | 0.7 | 5.9 | 10.1 | 18.4 | 3.0 | 13.6 | 27.1 | 1684.0 | 7052.6 | 15515.4 | 0.5 | 1.4 | 2.4 |
| 241 | 0.8 | 2.7 | 9.1 | 7.3 | 2.7 | 10.2 | 10.2 | 1396.3 | 5647.0 | 6399.1 | 0.3 | 1.2 | 1.0 |
| 242 | 0.9 | 3.0 | 5.8 | 11.8 | 3.2 | 7.2 | 18.9 | 3018.7 | 4015.6 | 10196.3 | 0.6 | 1.0 | 1.8 |
| 243 | 1 | 3.1 | 6.4 | 12.5 | 2.7 | 9.0 | 20.9 | 1725.5 | 4097.7 | 9698.1 | 0.6 | 1.3 | 2.1 |

|  |  | Ni |  |  | Cu |  |  | Zn |  |  | As |  |  |
| --- | --- | --- | --- | --- | --- | --- | --- | --- | --- | --- | --- | --- | --- |
| ID | Depth | > 250μm | 250-63μm | < 63μm | > 250μm | 250-63μm | < 63μm | > 250μm | 250-63μm | < 63μm | > 250μm | 250-63μm | < 63μm |
| 180 | 0.1 | 118958.3 | 109278.3 | 40141.4 | 7.4 | 12.3 | 6.0 | 27.6 | 44.3 | 17.1 | 24.8 | 39.8 | 19.4 |
| 181 | 0.2 | 115447.2 | 121142.4 | 139891.2 | 9.0 | 10.2 | 17.7 | 27.9 | 29.5 | 55.5 | 32.1 | 37.0 | 55.0 |
| 182 | 0.3 | 135546.3 | 68843.0 | 117550.5 | 10.8 | 5.5 | 14.5 | 38.3 | 17.2 | 45.4 | 22.2 | 10.6 | 27.6 |
| 183 | 0.4 | 117490.5 | 118115.5 | 120939.6 | 13.4 | 12.2 | 14.5 | 46.9 | 37.2 | 46.9 | 29.9 | 26.4 | 32.5 |
| 184 | 0.5 | 124602.8 | 123359.7 | 122234.1 | 45.3 | 11.9 | 14.0 | 38.3 | 38.7 | 46.0 | 30.4 | 30.4 | 35.4 |
| 185 | 0.6 | 116642.3 | 123938.1 | 127482.2 | 13.2 | 14.8 | 14.3 | 44.7 | 47.1 | 46.8 | 41.6 | 51.6 | 50.3 |
| 186 | 0.7 | 125171.1 | 122124.2 | 118011.9 | 332.8 | 17.8 | 271.5 | 55.3 | 65.8 | 64.8 | 54.1 | 82.7 | 80.4 |
| 187 | 0.8 | 125776.7 | 119845.0 | 28998.7 | 10.2 | 21.8 | 4.6 | 27.3 | 58.6 | 10.5 | 64.0 | 144.0 | 31.5 |
| 188 | 0.9 | 117957.1 | 122682.9 | 119113.2 | 21.4 | 24.2 | 21.2 | 105.8 | 67.2 | 65.0 | 79.9 | 88.7 | 85.3 |
| 189 | 1 | 127891.5 | 112045.6 | 87677.3 | 5.2 | 15.7 | 13.9 | 15.7 | 45.0 | 39.8 | 12.0 | 43.7 | 41.1 |
| 190 | 1.15 | 121798.5 | 115602.3 | 116273.4 | 2.2 | 8.2 | 19.3 | 9.2 | 27.0 | 54.4 | 1.6 | 4.7 | 9.8 |
| 191 | 1.25 | 117795.4 | 117234.4 | 119487.4 | 8.8 | 12.9 | 18.5 | 22.5 | 45.0 | 47.8 | 2.6 | 5.1 | 6.9 |
| 192 | 1.35 | 117673.6 | 111167.1 | 119719.3 | 9.1 | 11.5 | 466.6 | 29.7 | 34.6 | 81.4 | 4.8 | 5.9 | 9.2 |
| 193 | 1.4 | 124145.5 | 120853.4 | 120754.4 | 7.3 | 11.2 | 708.0 | 23.5 | 35.5 | 96.0 | 5.7 | 8.5 | 13.4 |
|  |  |  |  |  |  |  |  |  |  |  |  |  |  |
| 234 | 0.1 | 121088.9 | 131455.6 | 186891.7 | 4.8 | 11.8 | 18.4 | 16.4 | 42.3 | 50.1 | 8.4 | 24.7 | 31.4 |
| 235 | 0.2 | 122198.8 | 121232.8 | 193938.1 | 8.9 | 14.9 | 15.1 | 24.8 | 39.9 | 41.5 | 39.1 | 72.2 | 79.2 |
| 236 | 0.3 | 124247.3 | 131569.8 | 197212.3 | 3.1 | 14.7 | 14.8 | 6.0 | 33.5 | 37.2 | 8.9 | 44.9 | 54.4 |
| 237 | 0.4 | 129472.5 | 130849.6 | 186359.5 | 2.0 | 9.0 | 13.4 | 0.8 | 18.8 | 32.9 | 1.3 | 4.8 | 7.0 |
| 238 | 0.5 | 130882.2 | 124023.2 | 192347.0 | 1.3 | 6.1 | 10.2 | -0.1 | 12.8 | 26.0 | 1.0 | 4.6 | 6.8 |
| 239 | 0.6 | 125375.7 | 111806.9 | 195532.2 | 2.5 | 575.7 | 9.2 | 0.3 | 43.0 | 16.2 | 0.9 | 3.0 | 5.9 |
| 240 | 0.7 | 128928.8 | 136821.5 | 206645.5 | 1261.4 | 3.4 | 7.4 | 83.8 | 8.7 | 12.6 | 0.8 | 3.0 | 4.0 |
| 241 | 0.8 | 135112.6 | 119618.4 | 97301.7 | 1.7 | 3.1 | 2.6 | -0.2 | 4.1 | 2.0 | 0.8 | 2.8 | 1.8 |
| 242 | 0.9 | 124623.2 | 122212.1 | 190857.4 | 1.2 | 2.2 | 7.0 | -0.6 | 1.5 | 5.7 | 1.5 | 2.2 | 3.6 |
| 243 | 1 | 125117.6 | 121078.2 | 189606.4 | 1.3 | 2.9 | 8.6 | -0.9 | 1.6 | 5.0 | 1.0 | 2.0 | 3.1 |

|  |  | Se |  |  | Mo |  |  | Cd |  |  | Sb |  |  |
| --- | --- | --- | --- | --- | --- | --- | --- | --- | --- | --- | --- | --- | --- |
| ID | Depth | > 250μm | 250-63μm | < 63μm | > 250μm | 250-63μm | < 63μm | > 250μm | 250-63μm | < 63μm | > 250μm | 250-63μm | < 63μm |
| 180 | 0.1 | 1.5 | 1.4 | 0.6 | 0.3 | 0.3 | 0.1 | 5.2 | 0.1 | 0.0 | 0.1 | 0.1 | 0.0 |
| 181 | 0.2 | 1.4 | 1.4 | 2.0 | 0.2 | 0.2 | 0.5 | 0.0 | 0.1 | 0.1 | 0.1 | 0.1 | 0.1 |
| 182 | 0.3 | 1.9 | 0.9 | 1.7 | 0.2 | 0.1 | 0.2 | 0.0 | 0.0 | 0.1 | 0.1 | 0.0 | 0.1 |
| 183 | 0.4 | 2.0 | 1.6 | 1.6 | 0.2 | 0.2 | 0.3 | 0.1 | 0.0 | 0.0 | 0.0 | 0.0 | 0.1 |
| 184 | 0.5 | 1.9 | 1.5 | 2.1 | 0.2 | 8.1 | 0.2 | 0.1 | 0.2 | 0.0 | 0.2 | 0.1 | 0.1 |
| 185 | 0.6 | 1.6 | 1.6 | 1.7 | 0.2 | 0.3 | 0.2 | 0.0 | 0.2 | 0.0 | 0.1 | 0.1 | 0.1 |
| 186 | 0.7 | 1.7 | 2.0 | 1.8 | 0.2 | 0.3 | 0.3 | 0.1 | 0.1 | 0.1 | 1.1 | 0.1 | 0.9 |
| 187 | 0.8 | 1.7 | 1.7 | 0.4 | 0.2 | 0.3 | 0.1 | 0.0 | 0.1 | 0.0 | 0.1 | 0.1 | 0.0 |
| 188 | 0.9 | 2.1 | 2.0 | 1.9 | 0.3 | 0.3 | 0.4 | 0.1 | 0.1 | 0.1 | 0.1 | 0.1 | 0.1 |
| 189 | 1 | 1.5 | 1.7 | 1.5 | 0.1 | 0.2 | 0.2 | 0.0 | 0.1 | 0.0 | 0.0 | 0.1 | 0.1 |
| 190 | 1.15 | 1.3 | 1.4 | 1.9 | 0.1 | 0.2 | 0.3 | 0.0 | 0.0 | 0.1 | 0.0 | 0.0 | 0.0 |
| 191 | 1.25 | 1.6 | 2.0 | 2.1 | 0.2 | 0.3 | 0.2 | 0.0 | 0.1 | 0.1 | 0.0 | 0.0 | 0.0 |
| 192 | 1.35 | 1.6 | 1.8 | 2.2 | 0.3 | 0.3 | 0.3 | 0.1 | 0.1 | 0.1 | 0.0 | 0.0 | 1.7 |
| 193 | 1.4 | 1.9 | 2.1 | 2.4 | 0.2 | 0.2 | 0.6 | 0.0 | 0.0 | 0.0 | 0.0 | 0.0 | 1.4 |
|  |  |  |  |  |  |  |  |  |  |  |  |  |  |
| 234 | 0.1 | 1.2 | 1.9 | 1.5 | 0.1 | 0.2 | 0.2 | 0.0 | 0.1 | 0.0 | 0.1 | 0.0 | 0.0 |
| 235 | 0.2 | 1.3 | 1.7 | 1.3 | 0.2 | 0.3 | 0.2 | 0.0 | 0.0 | 0.0 | 0.1 | 0.0 | 0.1 |
| 236 | 0.3 | 1.2 | 1.8 | 1.5 | 0.1 | 0.3 | 0.2 | 0.0 | 0.1 | 0.0 | 0.0 | 0.7 | 0.0 |
| 237 | 0.4 | 1.1 | 1.6 | 1.6 | 0.1 | 0.2 | 0.4 | 0.0 | 0.0 | 0.0 | 0.0 | 0.0 | 0.0 |
| 238 | 0.5 | 1.2 | 1.4 | 1.7 | 0.1 | 0.2 | 0.2 | 0.0 | 0.0 | 0.0 | 0.0 | 0.0 | 0.0 |
| 239 | 0.6 | 0.9 | 1.3 | 1.6 | 0.1 | 0.2 | 0.2 | 0.0 | 0.1 | 0.0 | 0.0 | 1.7 | 0.0 |
| 240 | 0.7 | 1.1 | 1.4 | 1.4 | 0.2 | 0.2 | 0.1 | 0.0 | 0.0 | 0.0 | 2.5 | 0.0 | 0.0 |
| 241 | 0.8 | 1.2 | 1.2 | 0.7 | 0.1 | 0.2 | 0.1 | 0.0 | 0.0 | 0.0 | 0.0 | 0.0 | 0.0 |
| 242 | 0.9 | 0.9 | 1.1 | 1.2 | 0.2 | 0.1 | 0.2 | 0.0 | 0.0 | 0.0 | 0.0 | 0.0 | 0.0 |
| 243 | 1 | 1.0 | 1.3 | 1.2 | 0.1 | 0.1 | 0.2 | 0.0 | 0.0 | 0.0 | 0.0 | 0.0 | 0.0 |

|  |  | W |  |  | Pb |  |  | U |  |
| --- | --- | --- | --- | --- | --- | --- | --- | --- | --- |
| ID | Depth | > 250μm | 250-63μm | < 63μm | > 250μm | 250-63μm | < 63μm | > 250μm | 250-63μm |
| 180 | 0.1 | 0.1 | 0.0 | 0.0 | 6.5 | 12.1 | 6.4 | 0.8 | 1.2 |
| 181 | 0.2 | 0.0 | 0.1 | 0.1 | 9.0 | 11.0 | 18.5 | 0.9 | 1.1 |
| 182 | 0.3 | 0.1 | 0.0 | 0.0 | 13.8 | 6.3 | 17.4 | 1.6 | 0.7 |
| 183 | 0.4 | 0.1 | 0.0 | 0.0 | 18.2 | 18.1 | 19.3 | 1.7 | 1.5 |
| 184 | 0.5 | 0.0 | 0.1 | 0.1 | 17.1 | 14.5 | 17.7 | 1.4 | 1.6 |
| 185 | 0.6 | 0.0 | 0.0 | 0.0 | 14.5 | 16.4 | 16.3 | 1.5 | 1.8 |
| 186 | 0.7 | 0.1 | 0.0 | 0.1 | 40.0 | 18.6 | 39.6 | 1.1 | 1.7 |
| 187 | 0.8 | 0.0 | 0.1 | 0.0 | 10.4 | 23.0 | 5.2 | 1.0 | 2.0 |
| 188 | 0.9 | 0.0 | 0.1 | 0.0 | 22.9 | 23.8 | 21.6 | 2.1 | 2.3 |
| 189 | 1 | 0.0 | 0.1 | 0.0 | 5.6 | 19.1 | 15.6 | 0.6 | 1.8 |
| 190 | 1.15 | 0.0 | 0.0 | 0.1 | 2.8 | 8.8 | 17.5 | 0.3 | 1.4 |
| 191 | 1.25 | 0.0 | 0.0 | 0.0 | 7.3 | 12.4 | 13.6 | 1.2 | 2.6 |
| 192 | 1.35 | 0.0 | 0.0 | 0.0 | 9.6 | 11.5 | 55.3 | 1.9 | 2.5 |
| 193 | 1.4 | 0.0 | 0.0 | 0.0 | 8.5 | 12.2 | 76.3 | 1.7 | 2.6 |
|  |  |  |  |  |  |  |  |  |  |
| 234 | 0.1 | 0.0 | 0.1 | 0.0 | 4.8 | 15.6 | 20.2 | 0.5 | 1.4 |
| 235 | 0.2 | 0.0 | 0.0 | 0.0 | 12.4 | 21.9 | 23.5 | 1.0 | 2.1 |
| 236 | 0.3 | 0.0 | 0.0 | 0.0 | 4.0 | 19.2 | 22.1 | 0.3 | 1.5 |
| 237 | 0.4 | 0.0 | 0.0 | 0.0 | 2.0 | 10.1 | 15.1 | 0.2 | 1.0 |
| 238 | 0.5 | 0.0 | 0.0 | 0.0 | 1.7 | 8.5 | 13.9 | 0.2 | 1.2 |
| 239 | 0.6 | 0.0 | 0.0 | 0.0 | 1.4 | 53.4 | 13.8 | 0.1 | 0.5 |
| 240 | 0.7 | 0.0 | 0.0 | 0.0 | 103.2 | 6.0 | 12.3 | 0.1 | 0.4 |
| 241 | 0.8 | 0.0 | 0.0 | 0.0 | 1.1 | 5.4 | 5.4 | 0.1 | 0.3 |
| 242 | 0.9 | 0.0 | 0.0 | 0.0 | 1.5 | 4.6 | 10.1 | 0.1 | 0.3 |
| 243 | 1 | 0.0 | 0.0 | 0.0 | 1.6 | 5.3 | 9.7 | 0.1 | 0.3 |

**Table S3**: Principal component analysis of river-bank deposits for the Loddon catchment

| **Variable** | **PC1** | **PC2** | **PC3** | **PC4** |
| --- | --- | --- | --- | --- |
| Fe | 0.649 | 0.638 | 0.073 | -0.181 |
| Mn | 0.249 | 0.559 | -0.302 | 0.091 |
| Cu | 0.180 | 0.097 | 0.309 | 0.895 |
| Zn | 0.600 | 0.616 | 0.333 | -0.011 |
| As | 0.534 | -0.579 | 0.094 | 0.244 |
| Pb | 0.824 | -0.237 | 0.040 | -0.045 |
| Silt/Clay (%) | 0.604 | -0.668 | -0.140 | -0.099 |
| Fine sand (%) | -0.294 | 0.389 | -0.742 | 0.305 |
| Sand (%) | -0.450 | 0.331 | 0.778 | -0.076 |
| LOI | 0.443 | 0.523 | -0.214 | -0.085 |
| Total variance % | 26.9 | 24.8 | 15.3 | 10.2 |
| Cumulative variance % | 26.9 | 51.7 | 67.1 | 77.3 |

**Table S4**: Principal component analysis of two river-bank deposits (Back Eddington and Bridgewater) for the Loddon catchment

| **Variable** | **PC1** | **PC2** | **PC3** | **PC4** |
| --- | --- | --- | --- | --- |
| Fe | 0.973 | -0.001 | -0.095 | -0.134 |
| Mn | 0.557 | 0.782 | -0.035 | 0.102 |
| Cu | 0.081 | -0.107 | 0.955 | 0.243 |
| Zn | 0.941 | -0.080 | 0.064 | 0.230 |
| As | 0.664 | -0.638 | -0.263 | 0.037 |
| Pb | 0.603 | -0.227 | 0.753 | 0.096 |
| Mg | 0.886 | 0.412 | -0.049 | 0.094 |
| Al | 0.861 | 0.444 | -0.044 | -0.084 |
| Silt/Clay (%) | 0.673 | -0.701 | -0.130 | -0.116 |
| Fine sand (%) | -0.437 | 0.691 | 0.350 | -0.424 |
| Sand (%) | -0.349 | 0.362 | -0.240 | 0.826 |
| LOI | 0.592 | 0.611 | -0.110 | -0.128 |
| Total variance % | 46.6 | 24.4 | 14.8 | 8.8 |
| Cumulative variance % | 46.6 | 71.1 | 85.9 | 94.7 |

**References**

Shepard, F. P. (1954). "Nomenclature based on sand-silt-clay ratios." Journal of sedimentary Research 24(3): 151-158.
